# The culturable mycobiota of *Flabellia petiolata*: First survey of marine fungi associated to a Mediterranean green alga

**DOI:** 10.1371/journal.pone.0175941

**Published:** 2017-04-20

**Authors:** Giorgio Gnavi, Laura Garzoli, Anna Poli, Valeria Prigione, Gaëtan Burgaud, Giovanna Cristina Varese

**Affiliations:** 1 *Mycotheca Universitatis Taurinensis* (MUT), Department of Life Sciences and Systems Biology, University of Turin, Turin, Italy; 2 Université de Brest, EA 3882 Laboratoire Universitaire de Biodiversité et Ecologie Microbienne, Technopôle Brest-Iroise, Plouzané, France; Institute for Sustainable Plant Protection, C.N.R., ITALY

## Abstract

Algae-inhabiting marine fungi represent a taxonomically and ecologically interesting group of microorganisms still largely neglected, especially in temperate regions. The aim of this study was to isolate and to identify the culturable mycobiota associated with *Flabellia petiolata*, a green alga frequently retrieved in the Mediterranean basin. Twenty algal thalli were collected from two different sampling sites in the Mediterranean Sea (Elba Island, Italy). A polyphasic approach showed the presence of a relevant alga-associated mycobiota with 64 taxa identified. The fungal isolates belonged mainly to Ascomycota (61 taxa), while only three Basidiomycota were detected. The phylogenetic position of sterile mycelia and cryptic taxa, inferred on the basis of LSU partial region, highlighted the presence of putative new phylogenetic lineages within Dothideomycetes and Sordariomycetes. This work represents the first quali-quantitative analysis of the culturable mycobiota associated to a green alga in the Mediterranean Sea.

## Introduction

Oceans harbour a broad diversity of habitats and a huge diversity of prokaryotes but also of eukaryotic microorganisms, among which fungi are often dominant [1]. Marine fungi represent an ecological rather than a taxonomical defined group, comprising organisms belonging to different orders or phyla that share eco-physiological features. They have been retrieved from almost every kind of abiotic and biotic substrates, such as sediments, sponges, corals, echinoderms, vertebrates, algae, in a tremendous diversity of habitats ranging from coastal waters to the deep biosphere [2]. Albeit their diversity has recently been estimated to exceed 10,000 species/phylotype, a recent update indicated that only 1,112 species of marine fungi have been described, highlighting the gap of knowledge on marine fungi with almost 90% of the diversity to be described, mostly from uncharted marine environments [3]. In addition, basic knowledge on their distribution and ecological roles is still in its infancy [3–5].

Algae represent an important isolation source of marine fungi with almost one-third of all known marine fungal species associated with these organisms [2, 6]. Algae-inhabiting fungi represent a taxonomically diverse group of mutualists, endosymbionts, parasites, pathogens and saprobes, which are of evolutionary, ecological and economical interest [7, 8]. A number of studies have demonstrated that algae-inhabiting fungi were responsible for the production of many bioactive secondary metabolites, previously attributed to the host [9, 10]. Despite algal flora dominates marine habitats in temperate regions (9,200–12,500 described seaweeds), relatively few species have been investigated for the presence of an associated mycobiota; consequently further isolation efforts are required. Algicolous fungi associated to different seaweeds have been recently reviewed by Jones et al. [11] and Suryanarayanan [12].

*Flabellia petiolata* (Turra) Nizamuddin is a green alga commonly retrieved in the Mediterranean basin that belongs to the Udoteaceae family (Chlorophyta, Bryopsidales) [13]. *F*. *petiolata* colonises rocky and coral substrates of the sublittoral zone, often in association with other algae (e.g. *Dictyopteris* spp., *Dictyota* spp., *Dilophus* spp.). Moreover *F*. *petiolata* is one of the main components of the phytocoenoses associated with the endemic and endangered sea grass *Posidonia oceanica* [14, 15]. Compared to many other green algae, *F*. *petiolata* appears to be an interesting species, since antibacterial, antiviral, antimitotic, antifungal and cytotoxic activities have been detected in its raw extract [16]: whether the green alga or any associated organism produces biocides has never been clarified.

Despite its ecological and potential biotechnological value, *F*. *petiolata* has never been explored for its culturable mycobiota. This study aims (i) to isolate and identify marine fungi associated with *F*. *petiolata* and (ii) to create an exhaustive collection of fungal strains with putative future biotechnological applications.

## Material and methods

### Sampling procedures

**Fig 1:**
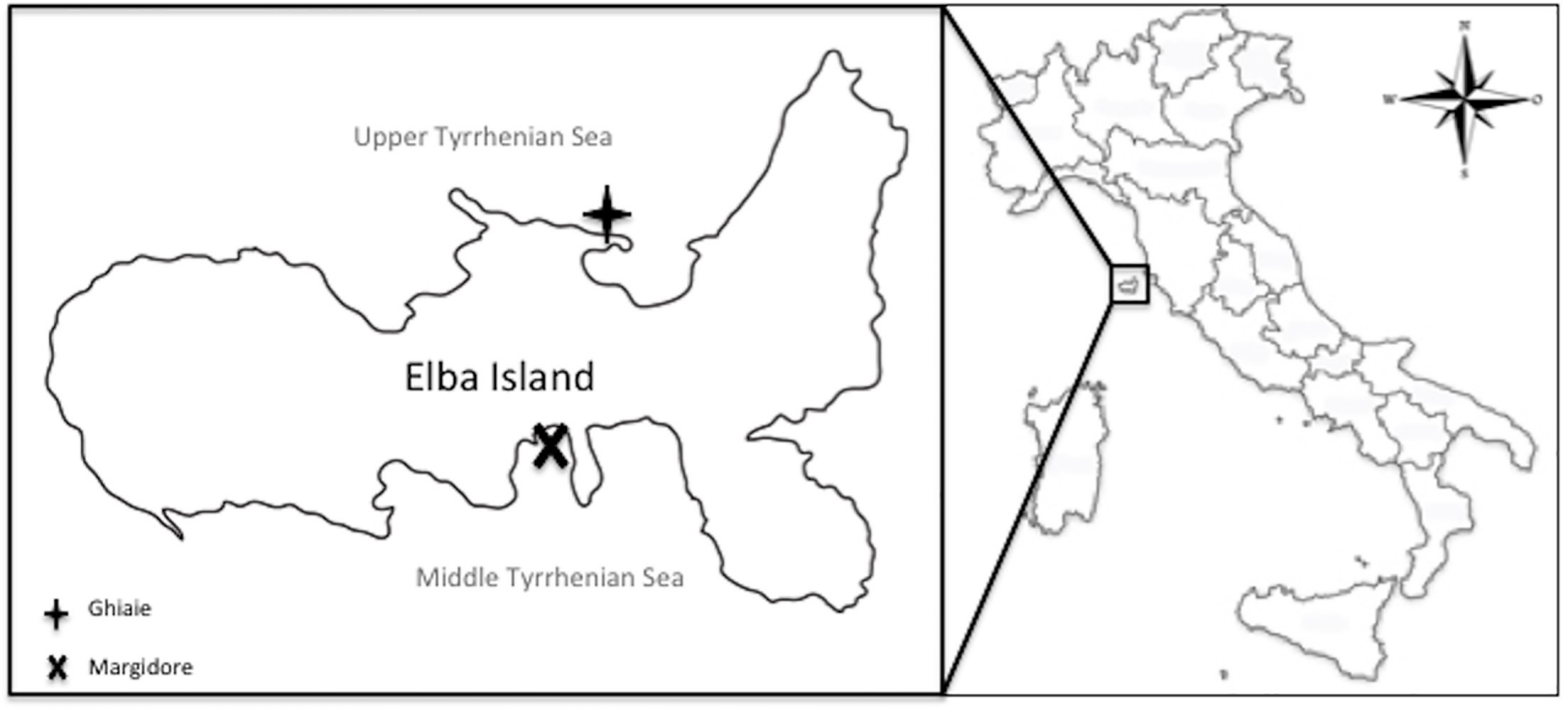
Sampling sites. Elba Island (Livorno), Tuscany, Tyrrhenian Sea (Mediterranean Sea) Italy.

Samples of *F*. *petiolata* were collected in March 2010 along the coasts of the Elba Island (Livorno, Italy) in the Tyrrhenian Sea (NW Mediterranean Sea). Two sampling sites, characterized by the presence of *P*. *oceanica* meadows associated with *F*. *petiolata*, were chosen: Ghiaie (UTM WGS84 42°49’04”N, 10°19’20”E) and Margidore (UTM WGS84 42°45’29”N, 10°18’24”E); depth ranged between 5 and 15 m below sea level (bsl) (Fig 1). A total of 20 algal thalli, 10 for each sampling site, were harvested. To avoid contaminations, algae were collected in sterile containers and maintained at 4°C during transportation. The samples were processed within 36 h from sampling. Specific permissions to operate in the protected area of “Le Ghiaie” (Ghiaie site) and to the freely accessible Margidore site were obtained by the port authority of Portoferraio (Livorno, Italy). Field study did not involve endangered or protected species.

### Fungal isolation

Each thallus was sonicated (30” each time) and serially washed (three times) in artificial sterilized SeaWater (SW, 3.4% w/v Sea Salt mix—Sigma-Aldrich, Saint Louis, USA—in ddH_2_O) to remove unrefined sediments. Then it was homogenized in 20 mL of sterile filtered seawater by means of a sterile device (Ultra-Turrax—IKA, Staufen, Germany). One mL of homogenate was plated in 12 cm diameter Petri dishes containing 30 mL of the following media: Corn Meal Agar SeaWater (CMASW) medium (17g CMA—Sigma-Aldrich, Saint Louis, USA—dissolved in 1 L of filtered SW) and Flabellia Agar SeaWater (FASW) medium (1g fw of *F*. *petiolata* in 100 mL of SW boiled for 30 minutes at 60°C and filtered; 18 g agar; SW up 1L). Each medium was autoclaved, supplemented with antibiotics (Gentamicin 80 mg/L, Piperacillin and Tazobactam 100 mg/L—Sigma-Aldrich, Saint Louis, USA) and further sterilized by filtration to prevent bacterial growth. Three replicates per medium and per sample were performed [17].

A total of 120 plates were incubated at 15°C for 15 days (spring average temperature of the Elba Island submerged meadows at depths between 5 and 15 m bsl) to allow the isolation of psychrotolerant or psychrotrophic fungi. Plates were subsequently placed at 24°C for 45 days to allow the development of mesophilic colonies including the slow-growing ones. The number of colony forming units per gram of dry weight of each algal thallus (CFU/g dw) was recorded. For filamentous fungi, CFU refer to individual colonies originating from a single or a mass of cells or spores/conidia. Strains from each fungal morphotype and from each sampling site were isolated in pure culture and preserved at the *Mycotheca Universitatis Taurinensis* (MUT, http://www.mut.unito.it/en; MUT codes are reported in the Results section).

### Fungal identification

A polyphasic approach was employed to identify the isolated strains. First, fungi were identified according to their macroscopic, microscopic and physiological features (S1 Fig) on the basis of specific taxonomical keys, following the indications provided from Dictionary of the Fungi [18] and from the Mycobank databases (http://www.mycobank.org/). Subsequently, molecular analyses were performed by sequencing specific genomic DNA regions.

### DNA extraction and amplification

Genomic DNA was extracted following a modified protocol of Cubero et al. [19]. In detail, 100 mg of mycelium were gently scraped from an agar petri dish, placed in a 2 mL Eppendorf tube and disrupted in a MM400 tissue lyzer (Retsch GmbH, Haan, Germany). A volume of 0.5 mL of pre-warmed extraction buffer (1% w/v CTAB; 1M NaCl; 100 mM Tris; 20 mM EDTA; 1% w/v polyvinyl polypyrolidone, PVPP added to the buffer immediately prior to use—Sigma-Aldrich, Saint Louis, USA) was added to the ground material. Samples were vortexed and heated in a water bath for 30 min at 60°C. Following, one volume of chloroform: isoamyl alcohol (24:1 v/v—Sigma-Aldrich, Saint Louis, USA) was added, samples were vortexed and centrifuged for 3 min at 10,000 g at room temperature. The upper aqueous phase was collected in a new tube and two volumes of precipitation buffer (1% w/v CTAB; 50 mM Tris-HCl; 10 mM EDTA; 40 mM NaCl—Sigma-Aldrich, Saint Louis, USA) were added. The mixture was vortexed and centrifuged for 10 min at 14,000 g at room temperature. Supernatant was discarded, the pellet was collected and resuspended in 350 μL of 3 M Sodium Acetate (CH_3_COONa—Sigma-Aldrich, Saint Louis, USA), to which one volume of chloroform: isoamyl alcohol (24:1) was added. Samples were vortexed and centrifuged for 3 min at 10,000 g at room temperature. The upper phase was placed in a new tube and 660 μL of isopropanol were added prior to incubation at -20°C for 20 min. The final pellet was collected by centrifugation for 10 min at 14,000 g at 4°C. Finally, the pellet was washed with 1 mL of 70% ethanol and recollected by centrifugation for 2 min at 14,000 g at 4°C. The pellet was dried at 40°C and subsequently resuspended in 60 μL of TE buffer (10 M Tris pH 7.4, 1 mM EDTA—Sigma-Aldrich, Saint Louis, USA).

The quality and quantity of extracted DNA was measured by using NanoDrop 1000 (Thermo Scientific, Wilmington, USA). DNAs were stored at -20°C.

Specific markers were amplified in a Biometra TGradient Thermocycler (Biometra, Göttingen, Germany) as follows. PCR mixture consisted of 5 μL 10x PCR Buffer (15 mM MgCl_2_, 500 mM KCl, 100 mM Tris-HCl, pH 8.3) 0.4 mM MgCl_2_, 0.2 mM each dNTP, 1 μM each primer, 2.5 U Taq DNA Polymerase (all reagents were supplied by Sigma-Aldrich, Saint Louis, USA), 40–80 ng DNA, in 50 μL final volume. For more details about PCR cycles, see the S2 Table.

The nr DNA partial regions (ITS or LSU and SSU when necessary) were amplified using the universal primers ITS1/ITS4 [20, 21], LR0R/LR7 [22], and NS1/NS4 [23]. For the strains morphologically identified as *Cladosporium* spp. it was necessary to amplify the Actin gene using primers ACT512F/ACT783R [24]. For those strains identified as *Penicillium* spp. the β-tubulin gene was amplified using the primer pair Bt2a/Bt2b [25]. PCR products were purified and sequenced at Macrogen Europe (Amsterdam, The Netherlands). Consensus sequences were obtained by using Sequencer 5.0 (Gene Code Corporation, http://www.genecodes.com). Taxonomic assignments were inferred by querying with the Blastn algorithm (default setting), hosted at NCBI (National Center for Biotechnology Information—http://www.ncbi.nlm.nih.gov) the newly generated sequences against the nucleotide database of NCBI (GenBank). Pairwise alignments were also performed at http://www.cbs.knaw.nl against the CBS-Knaw Fungal Biodiversity Centre (Centraalbureau voor Schimmelcultures) database. Similarity values equal or higher than 98% (e-value > e^-100^) were considered credible and the results were confirmed morphologically. Sequences related to fungi isolated in this study were deposited at the NCBI database (GenBank accession no. KP671714—KP671750; KR014346—KR014380; KT313376—KT313393; KT587307—KT587334; KU315005—KU315009; KX988016—KX988018; KY081460—KY081463; KY081637). When low sequence similarity (< 98%) did not allow genus and/or species determination, or when the strain remains sterile in pure culture, the taxonomic position was inferred through phylogenetic analysis. A full phylogenetic analysis was performed on LSU sequences, since comparable ITS and SSU sequences of fungi studied in this paper are rare in public databases and/or poorly informative. Four sequences datasets were properly composed following Suetrong et al. [26], and Hyde et al. [27] for Pleosporales (127 sequences) and Capnodiales (85 sequences), Wang et al. [28, 29] and Nekoduka et al. [30] for Leotiomycetes (71 sequences), and Tang et al., [31] for Sordariomycetes (165 sequences). The complete dataset is provided in Supporting Information (see S1 Dataset). Alignments were generated using MUSCLE, implemented in MEGA 6.0 (Molecular Evolutionary Genetics Analysis, [32]), and manually refined (number of characters were 733, 776, 782, 814 for Leotiomycetes, Pleosporales, Sordariomycetes, Capnodiales, respectively). Phylogenetic analyses were performed using both Bayesian Inference (BI; MrBayes 3.2.2; four incrementally heated simultaneous Monte Carlo Markov Chains (MCMC), run over 10 million generations, under GTR + Γ evolutionary model) and Maximum Likelihood (ML; RAxML v.7.3.2; 1,000 bootstraps replicates using the GTRGAMMA algorithm) approaches, as extensively described in Gnavi and collaborators [33]. Since both phylogenetic models yielded the same topology only the Bayesian trees were displayed. Bayesian Posterior Probability (BPP) values over 0.70 are reported in the resulting trees.

### Statistical analysis

Statistical analyses were performed using PRIMER 7.0 (Plymouth Routines In Multivariate Ecological Research [34]). The biodiversity within sampling sites was estimated by calculating Shannon-Weaver’s index (H’), Gini-Simpson’s index (1-Lambda) and Pielou’s evenness (J’) on presence/absence matrix (S3 Table). The difference between fungal abundance at the different locations or on the isolation media was evaluated with PAST 3.x software [35] using F-test (p ≤ 0.05). The Non-Metric Multi Dimensional Scaling (NMDS) analysis was performed in R (Vegan package) [36].

## Results

### Quantitative analysis

All the thalli of *F*. *petiolata* led to the growth of fungal isolates. The average fungal abundance (CFUg^-1^dw) of the 10 thalli from each site ranged between 4.8 x 10^2^ CFU g^-1^dw and 1.3 x 10^3^ CFU g^-1^dw (Table 1). The CMASW medium led to a higher fungal load compared to FASW. Most of the isolates required specific media and incubation temperatures: 28 taxa were exclusively isolated from CMASW, 30 from FASW and only 6 were isolated from both media. Ten taxa grew exclusively at 15°C, 50 were isolated only at 25°C, while the remaining four were retrieved in both conditions (Table 1). All the biodiversity indexes used were similar in the two sampling sites (Table 2).

**Table 1:** Fungal load and number of fungal entities isolated from *F*. *petiolata* thalli in different sites, different media and incubation temperatures.

sites	Ghiaie		Margidore	
media	FASW	CMASW	FASW	CMASW
**CFU/g dw ± SE**	5.4·10^2^± 2.4·10^1^ **a**	1.1 10^3^± 3.5·10^1^**b**	4.8·10^2^± 2.0·10^1^**a**	1.3·10^3^± 3.8·10^1^**b**
**Exclusive taxa (per medium)**	17 (0)	11 (2)	14 (3)	18 (5)
**Exclusive taxa (per site)**	28		31	
**Total taxa (per site)**	33		36	

Different lowercase letters indicate significant difference (p ≤ 0,05, F-test) among the load on the same medium obtained in different sites. In brackets taxa isolated exclusively at 15°C. FASW, Flabellia Agar Sea Water; CMASW, Corn Meal Agar Sea Water; CFU, Colony-Forming Unit; dw, dry weight; SE, Standard Error.

**Table 2:** Biodiversity values at the two sampling sites.

sites	taxa	individuals	H' (log e)	1-Lambda	J'
Ghiaie	33	44	3.37	0.98	0.97
Margidore	36	48	3.38	0.99	0.95

Shannon-Weaver’s index (H’), Gini-Simpson’s index (1-Lambda) and Pielou’s evenness (J’).

### Fungal diversity

**Fig 2:**
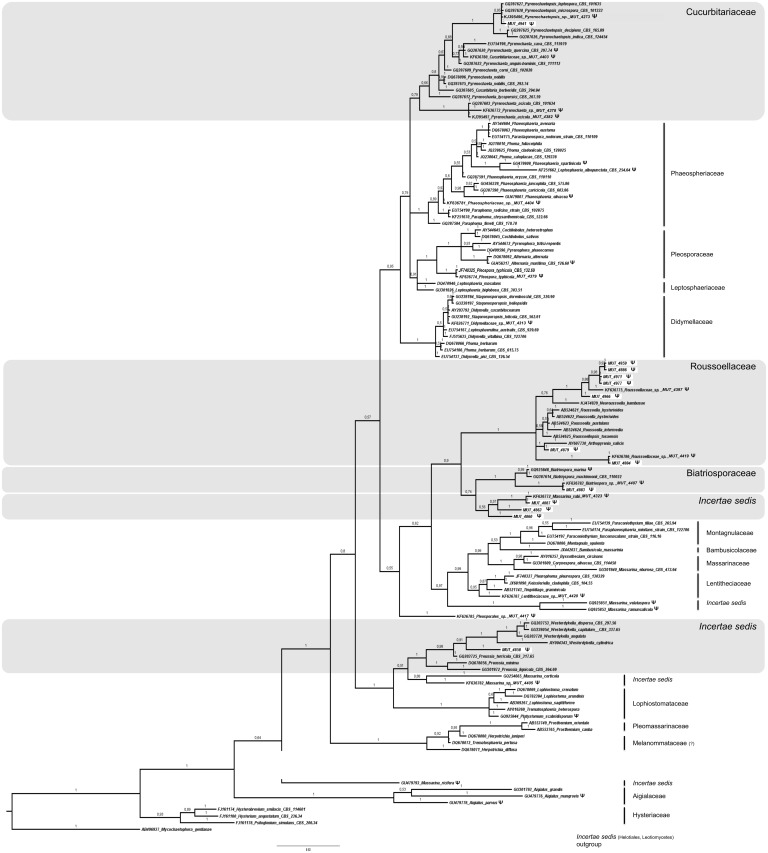
Bayesian phylogram of Pleosporales (Dothideomycetes) based on rDNA large subunit (LSU). Branch numbers indicate BPP over 0.70; ML bootstrap > 50%. Thirteen fungal isolates (indicated as MUT) are included. Strains from marine sources are labelled with symbol **Ψ**. Bar = expected changes per site (0.03).

**Fig 3:**
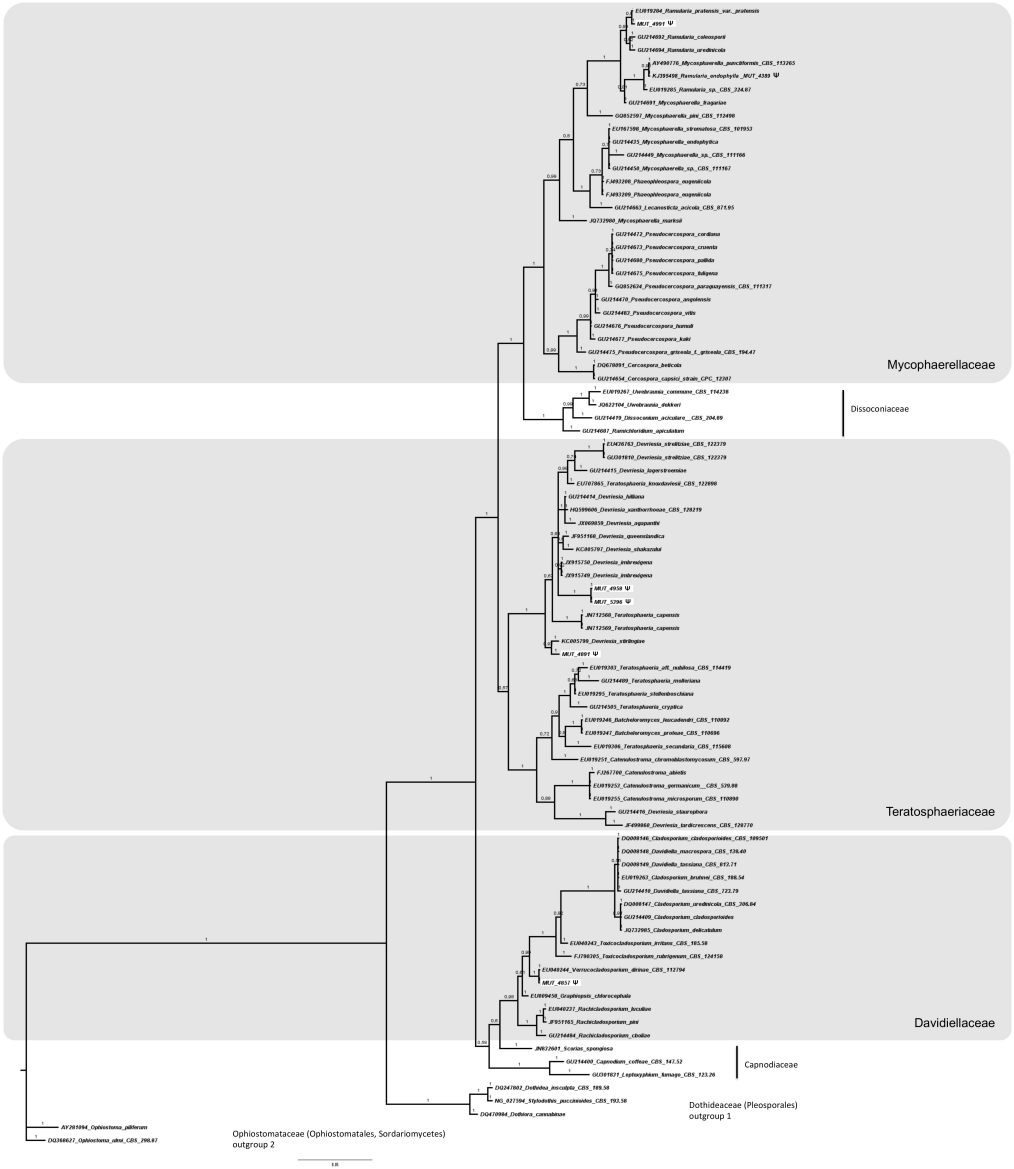
Bayesian phylogram of Capnodiales (Dothideomycetes) based on rDNA large subunit (LSU). Branch numbers indicate BPP over 0.70; ML bootstrap > 50%. Five fungal isolates (indicated as MUT) are included. Strains from marine sources are labelled with symbol **Ψ**. Bar = expected changes per site (0.05).

**Fig 4:**
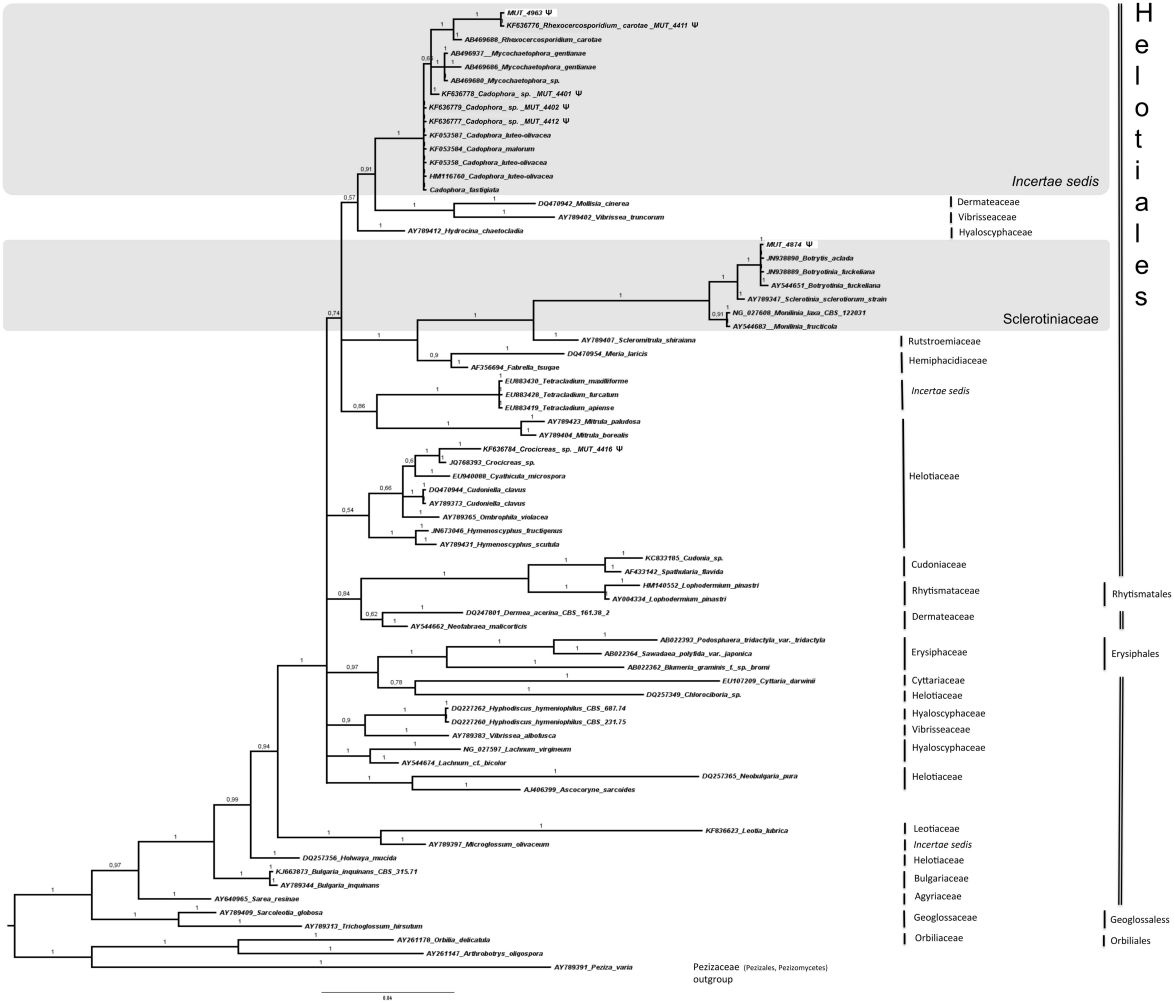
Bayesian phylogram of Leotiomycestes based on rDNA large subunit (LSU). Branch numbers indicate BPP over 0.70; ML bootstrap > 50%. Two fungal isolates (indicated as MUT) are included. Strains from marine sources are labelled with symbol **Ψ**. Bar = expected changes per site; value (0.04).

**Fig 5:**
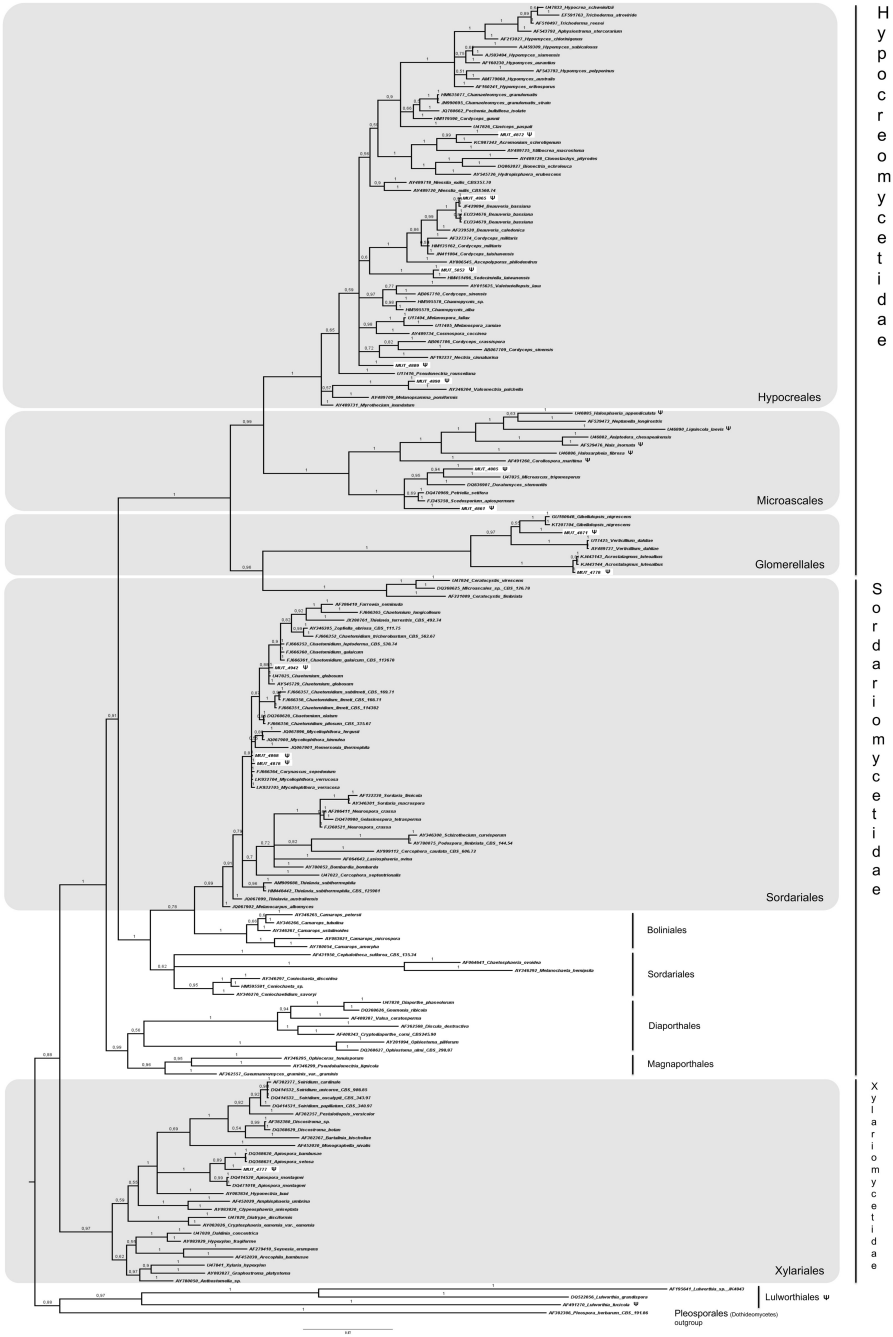
Bayesian phylogram of Sordariomycestes based on rDNA large subunit (LSU). Branch numbers indicate BPP over 0.70; ML bootstrap > 50%. Thirteen fungal isolates (indicated as MUT) are included. Strains from marine sources are labelled with symbol **Ψ**. Bar = expected changes per site (0.07).

A total of 143 fungal isolates, belonging to 64 taxa, were detected (Table 3). Since 23% of the isolates remained sterile in pure culture and sequence similarity through BLASTn analysis did not allow genus and/or species determination, a phylogenetic analysis based on LSU partial region was used to provide a valid classification. Both phylogenetic models yielded the same topology; therefore, only the Bayesian trees with BPP values are shown (Figs 2–5). In detail, the phylogenetic analysis showed that 13 strains were affiliated to the Pleosporales order (Dothideomycetes, Fig 2), 5 strains grouped in the Capnodiales (Dothideomycetes, Fig 3), 2 strains fell within the Helotiales (Leotiomycetes, Fig 4), and 13 within Sordariomycetes (Fig 5).

**Table 3:** Fungal entities isolated from *F*. *petiolata*: culture media, incubation temperature, area of sampling and accession numbers of the obtained sequences.

MUT CODE	Taxa	Isolation media	Incubation temperature		Sampling area		GenBank accession number				
			15°C	25°C	Ghiaie	Margidore	ITS	LSU	SSU	ACT	TUBC
**Agaricomycetes**											
4775	* Coprinellus sp.	FASW		x		+	KR014370	KP671736			
4993	* Peniophora sp.	FASW		x	+		KR014375	KP671738	KT587326		
4875	* Schizophyllum commune Fr.	CMASW		x		+	KX988018				
**Dothideomycetes**											
4772	***Alternaria alternata*** (Fr.) Keissl.	FASW		x		+	KX988016				
5071											
4879	***Arthopyrenia salicis*** A. Massal.	FASW		x		+	KR014347	KP671722			
4976	***Aureobasidium pullulans*** (de Bary) Arna.	CMASW		x		+	KR014373	KP671737	KT587333		
4883	* Biatriospora sp.	FASW		x		+	KR014352	KP671728	KT587328		
4774	***Cladosporium allicinum*** Bensch, & Crous	FASW		x	+					KU315005	
4985	***Cladosporium cladosporioides*** (Fresen) V.	CMASW, FASW		x	+					KU315007	
4996										KU315008	
5402											
4989	***Cladosporium herbarum*** (Pers.) Link	FASW		x		+				KY081637	
4776	***Cladosporium sphaerospermum*** Penz.	CMASW, FASW	x	x	+	+				KU315006	
5002										KU315009	
5004											
4891	* Devriesia sp.	FASW		x	+		KR014372	KP671742	KT587311		
4887	* Massarina rubi (Fuckel) Sacc.	FASW		x		+	KR014359	KP671721	KT587318		
4860	* Massarina sp.1	CMASW		x		+	KR014362	KP671730	KT587325		
4863	* Massarina sp.2	CMASW		x		+		KP671719	KT587316		
4941	* Pyrenochaetopsis sp.	CMASW	x			+	KR014354	KP671715	KT587320		
4991	* Ramularia eucalypti Crous	FASW		x	+		KR014378	KT313376			
4884	* Roussoellaceae sp. **1**	FASW		x	+			KP671726	KT587329		
4859	* Roussoellaceae sp. **2**	CMASW	x		+		KR014355	KP671716	KT587315		
4886	* Roussoellaceae sp. **3**	CMASW		x	+		KR014358	KP671720	KT587317		
4966	* Roussoellaceae sp. 4	CMASW		x		+	KR014366	KP671740	KT587309		
4971	* Roussoellaceae sp. 5	CMASW	x	x	+		KR014367	KP671734	KT587331		
4977	* Roussoellaceae sp. 6	CMASW		x	+			KP671748			
4858	* Sporormiaceae sp.	FASW		x		+		KP671731	KT587313		
4958	* Teratosphaeriaceae sp. 1	CMASW		x	+		KR014353	KP671744	KT587330		
5396	* Teratosphaeriaceae sp. 2	CMASW		x	+		KR014379	KT313377			
4857	*Verrucocladosporium dirinae K. Schub., Aptroot & Crous	FASW		x	+		KR014361	KP671739	KT587307		
**Eurotiomycetes**											
5408	* Herpotrichiellaceae sp.	FASW		x	+		KR014371	KP671741			
4979	* Knufia petricola (U. Wollenzien & de Hoog) Gorbushina & Gueidan	FASW		x	+		KR014376	KP671749			
4962	***Penicillium antarcticum*** Hocking & McRae	CMASW, FASW	x	x	+	+					KT313389
4967											
4970											KT313390
4973											
4974											KT313391
4980											
4990											
4994											
4997											
5000											KT313392
4960	***Penicillium atramentosum*** Thom	CMASW		x	+						
4965	***Penicillium brevicompactum*** Dierckx	FASW	x			+					KT313384
4987											
5397	***Penicillium chrysogenum*** Thom	FASW		x	+						
4856	***Penicillium commune*** Thom	CMASW		x		+					KT313385
4968											KT313381
5001											KT313393
5399											KT313383
4964	***Penicillium crustosum*** Thom	CMASW		x	+	+					KT313380
4984											KT313378
4877	***Penicillium expansum*** Link	CMASW	x			+					KT313388
4972	***Penicillium palitans*** Westling	CMASW		x		+					KT313379
4983	***Penicillium simplicissimum*** (Oudem.) Thom	FASW		x	+						
4978	***Penicillium solitum*** Westling	CMASW		x		+					KT313382
4862	***Penicillium* sp.**	FASW		x	+						KT313386
4870	***Talaromyces variabilis*** (Sopp) Samson, Yilmaz, Frisvad & Seifert	CMASW		x	+						KT313387
4888	* Trichomeriaceae sp.	FASW		x	+		KR014348	KP671723			
**Leotiomycetes**											
4874	***Botrytis cinerea*** Pers.	CMASW	x			+	KR014349	KP671724	KT587323		
4963	* Rhexocercosporidium carotae (Årsvoll) U. Braun	CMASW		x		+	KR014374	KP671743	KT587310		
**Sordariomycetes**											
4780	***Acremonium breve*** (Sukapure & Thirum.) W. Gams	CMASW	x			+	KY081463				
4975											
4872	* Acremonium sclerotigenum (Moreau & R. Moreau ex Valenta) W. Gams	FASW		x		+	KR014351	KP671727	KT587327		
4779	***Acremonium tumulicola*** Kiyuna, An, Kigawa & Sugiyama	FASW	x			+	KY081462				
4778	***Acrostalagmus luteoalbus*** Gams & Schroers	CMASW, FASW	x	x	+	+		KP671745	KT587308		
4783											
5047											
4777	***Apiospora montagnei*** Sacc.	FASW		x	+	+		KP671750			
4992											
4986	***Arthrinium mari*** Larrondo & Calvo	CMASW, FASW		x		+	KY081460				
4995											
4999	***Arthrinium phaeospermum*** (Corda) M. B. Ellis	CMASW	x		+		KY081461				
4865	***Beauveria bassiana*** (Bals.-Criv.) Vuill.	CMASW		x		+	KR014380	KP671729			
4942	* Chaetomium globosum Kunze	FASW		x	+		KR014363	KP671732	KT587334		
4781	***Emericellopsis minima*** Stolk	FASW		x	+		KR014377				
4981											
4982											
4871	* Gibellulopsis nigrescens (Pethybr.) Zare, W. Gams & Summerb.	CMASW		x	+		KR014364	KP671747	KT587321		
4855	***Gliomastix masseei*** (Sacc. & Trotter) Matsush.	FASW		x		+	KX988017				
4889	* Hypocreales sp.	FASW		x		+	KR014350	KP671725	KT587324		
4861	* Microascaceae sp.	CMASW		x		+	KR014360	KP671746	KT587322		
4864	***Microascus cirrosus*** Curzi	FASW		x	+				KT587314		
4885	* Microascus trigonosporus C.W. Emmons & B.O. Dodge	FASW	x			+	KR014356	KP671717	KT587319		
4868	* Myceliophthora verrucosa (Stchigel, Cano & Guarro) van den Brink & Samson	CMASW, FASW		x	+		KR014346	KP671714			
4878							KR014365	KP671733	KT587332		
4998	***Sarocladium strictum*** (W. Gams) Summerb.	CMASW	x			+					
5053	* Sedecimiella taiwanensis K.L. Pang, Alias & E.B.G. Jones	CMASW		x	+		KR014368	KP671735			
4890	* Valsonectria pulchella Speg.	CMASW		x		+	KR014357	KP671718	KT587312		

Sterile mycelia (*) underwent phylogenetic analyses; CMASW, Corn Meal Agar Sea Water; FASW, Flabellia Agar SeaWater; Sequence markers: ITS, Internal Transcribed Spacer; LSU, Large ribosomal SubUnit; SSU, Small ribosomal SubUnit; ACT, actin; TUB; β-tubulin.

Within Pleosporales, MUT 4941 was identified as *Pyrenochaetopsis* sp., MUT 4859, 4886, 4971, 4977, 4966 clustered with *Neoroussoella bambusae* (Roussoellaceae family), MUT 4879 as *Arthopyrenia salicis*, MUT 4884 as Roussoellaceae sp., MUT 4883 as *Biatriospora* sp., MUT 4887 as *Massarina rubi*, MUT 4863 and MUT 4860 as *Massarina* sp., and MUT 4858 was assigned to Sporormiaceae (Fig 2).

As for Capnodiales, MUT 4991 was identified as *Ramularia eucalypti*, MUT 4958 and 5396 clustered in the Teratosphaeriaceae family; MUT 4891 was affiliated to *Devriesia* genus, close to *Devriesia strelitziae*, and MUT 4857 as a *Verrucocladosporium dirinae* strain (Fig 3).

With respect to Helotiales, MUT 4963 was identified as *Rhexocercosporidium carotae*, while MUT 4874 was assigned to *Botrytis cinerea* (Fig 4).

Finally, thanks to the phylogenetic analyses, almost all Sordariomycetes were identified at species level: *Beauveria bassiana* (MUT 4865), *Acremonium sclerotigenum* (MUT 4872), *Sedecimiella taiwanensis* (MUT 5053), *Valsonectria pulchella* (MUT 4890), *Microascus trigonosporum* (MUT 4885), *Acrostalagmus luteoalbus* (MUT 4778), *Gibellulopsis nigrescens* (MUT 4871), *Chaetomium globosum* (MUT 4942), *Myceliophthora verrucosa* (MUT 4868 and 4878) and *Apiospora montagnei* (syn. *Arthrinium arundinis*, MUT 4777). Moreover, MUT 4889 was identified as Hypocreales sp. and MUT 4861 clustered within the Microascaceae (Fig 5).

According to these analyses, identification was possible at species level for 17 taxa and at genus level for 5 taxa; the remaining cryptic entities (12) were assigned to orders and families on the basis of clade similarities (Table 3).

At a broader scale, almost all taxa (61) belong to Ascomycota (24 Dothideomycetes, 15 Eurotiomycetes, 2 Leotiomycetes, 20 Sordariomycetes) and 3 to Basidiomycota (Agaricomycetes) (Table 3).

Although the biodiversity indexes were comparable, the isolated mycobiota associated to *F*. *petiolata* was different in the two sites: 31 taxa were isolated exclusively from Margidore, 28 from Ghiaie and only 5 taxa were recorded in both areas. *A*. *luteoalbus*, *C*. *cladosporioides*, *E*. *minima* and *M*. *verrucosa* were the most frequent taxa in in Ghiaie site, while *A*. *phaeospermun* and *P*. *commune* were the most frequent taxa in Margidore samples. However, the NMDS analysis (S2 Fig) revealed that this dissimilarity can not be ascribed to a site effect, but to a high intragroup variability. In fact more than 80% of the isolated taxa were retrieved only in individual thalli.

## Discussion

The aim of this study was to describe, for the first time, the culturable mycobiota associated with the green alga *F*. *petiolata* in the Mediterranean Sea. Although the approach employed does not fully unfold the whole fungal biodiversity, a quali-quantitative analysis of what we thought to be an exhaustive collection of marine fungal isolates was performed.

### Abundance of *F*. *petiolata*-inhabiting fungi

Few species belonging to Chlorophyta have been previously investigated for their mycobiota; those previous studies showed low fungal diversity associated to Chlorophyta, with an average of 10–20 fungal taxa from each algal species [11, 37, 38]. According to Zuccaro and Mitchell [38], the short life cycle of some of the green algal species and the peculiar slow growth of their endosymbionts could partly explain the low fungal diversity harboured by green algae. Nevertheless, the present survey demonstrated that *F*. *petiolata* supports a relevant associated mycobiota with a high fungal biodiversity (64 taxa isolated). The fungal abundance and species richness recorded on this alga are comparable to those usually found on brown and red seaweeds, which are considered to be the richest in terms of fungal diversity [7, 8, 37, 38]. The high number of taxa recorded is certainly due to the isolation procedure, which allowed the isolation of many species never recorded before in the Mediterranean Sea. Only few species were isolated on both media/temperatures, suggesting that most of them need specific growth requirements. The use of media/temperatures mimicking the natural environment, allowed the isolation of species that may be intimately associated with their host. This is the case of the lichenicolous species *Verrucocladosporium dirinae* [39], isolated only from FASW, and the cryptic Roussoellaceae strains isolated exclusively from CMASW at 15°C. Thus, the use of different media and incubation temperatures undoubtedly maximized the number of isolates and allowed to reveal between 7 and 14 times more fungal isolates than previously observed on other green algae [11, 12, 37]. However, a poor overlap was observed between the mycobiota of the two sampling sites suggesting that the overall culturable fungal diversity associated to *F*. *petiolata* is far from being fully resolved. A statistical analysis (NMDS, S2 Fig) revealed a huge intragroup variability (among fungal isolates of each thallus); consequently, it is not possible to detect any significant difference between the two diverse sites. Intriguingly, thallus S19 is clearly different from the others. This could be due to a peculiar association and/or absence of taxa in this sample. In addition, by inspecting the rarefaction curves relative to Ghiaie and Margidore (data not shown), it was clear that the saturation was far from being achieved: a much higher number of thalli would be necessary to estimate the richness of the culturable mycobiota, leading to a clearer, precise and more complete view of the biodiversity occurring. In particular, Ghiaie site is located in a marine protected area on the northern shore of the island whose seabed is mainly composed of rocks alternating with limestone gravel. Margidore site is instead located on southern shore, its bottom is a heterogeneous substrate formed by serpentinite, gabbros, diabase and is subjected to an intense anthropic disturbance [40], that may explain the higher fungal load retrieved in this area. In conclusion, we hypothesize that *F*. *petiolata* mycobiota could be affected by several abiotic factors including hydrodynamic force, geochemical substrate composition and anthropic disturbance.

### Ubiquitous *vs*. host-specific fungi

Likewise Suryanarayanan et al. [37] who analysed the fungal communities associated with six green algal species (*Caulerpa* spp., *Halimeda macroloba* and *Ulva* spp.), we observed that the mycobiota of *F*. *petiolata* includes few dominant species (*i*.*e*. *P*. *antarcticum*) and many rare/occasional ones. Unlike Garzoli and collaborators [41] who demonstrated high host specificity for the red alga *Asparagopsis taxiformis* in the Mediterranean Sea, *F*. *petiolata* appeared to be an easy substrate to colonize, as clearly highlighted by the high fungal biodiversity retrieved. This divergence in “substrate specificity” may be due to the different metabolites produced by red and green algae in response to different environmental and physical conditions [42]. For instance, the red alga *A*. *taxiformis*, as well as other red and brown algae [43], is well known for the production of several halogenated biocides [44] which can be involved in limiting the substrate colonization. On the contrary, till now, no antimicrobial compound has been identified in *F*. *petiolata* [45].

### Diversity and putative ecological roles of algae-inhabiting fungi

Ascomycota was found to be the most common phylum, confirming that, in the marine environment, algae-inhabiting fungi are mostly affiliated to the ascomycetes [4]. On the contrary, basidiomycetes appear to be rare, probably due to their inability to colonize algae. In fact, in algal thalli, lignin, the eligible substrate for basidiomycetes, is absent and is replaced with a high concentration of cellulose [2]. Only three basidiomycetes have been retrieved here, *i*.*e*. *Coprinellus* sp., *Peniophora* sp. and *Schizophyllum commune*. A strain of *Coprinellus* (*C*. *radians*) was already isolated from the zoanthid *Palythoa haddoni* [46] and *S*. *commune* was already detected in association with *P*. *oceanica* [47] and mangroves [48] (S1 Table). Finally, different fungal strains identified as *Peniophora* sp. were recently retrieved from an oil polluted marine site in the Mediterranean Sea [49]. Interestingly, the isolation of species belonging to the genera *Peniophora* and *Schizophyllum* from a cellulose substrate, such as *F*. *petiolata*, is in line with recent observations that demonstrated the ability of these basidiomycetes to produce cellulolytic enzymes [50, 51].

Regarding Ascomycota phylum, the most representative classes were Dothideomycetes and Sordariomycetes, followed by Eurotiomycetes (Table 3). This is in agreement with a recent publication by Jones and Pang [2], who described Dothideomycetes and Sordariomycetes as the most diffuse organisms (in terms of taxa) in these environments.

The high number of Dothideomycetes isolated from *F*. *petiolata* (38%) is not surprising. Species belonging to this class occur on a wide range of aquatic and marine substrata as mangrove wood, twigs and leaves, sea and marsh grasses [26, 27] and can be found in association with brown and red seaweeds [11]. Pleosporales is the largest order in the Dothideomycetes, comprising a quarter of all dothideomycetous species that occur in various habitat as epiphytes, endophytes or parasites of living leaves or stems, hyperparasites on fungi or insects, lichenized, or saprobes of dead plant stems, leaves or bark [52]. The phylogenetic analysis of pleosporalean sterile mycelia isolated from *F*. *petiolata* highlights the presence of a relevant number of strains that may represent entities never described before. Within Rousoellaceae, two new clades of marine origin were identified: (i) MUT 4859, 4886, 4966, 4971 and 4977 formed a distinct clade together with a strain isolated from *P*. *oceanica* [33], close to *Neoroussoella bambusae* (a monotypic genus described by Liu et al. [53]), and may represent a new species of the same genus; (ii) the other well supported clade included MUT 4884 and another strain isolated from *P*. *oceanica* [33], both from Mediterranean Sea. Within Biatriosporaceae, a *Biatriospora* sp. well supported clade was identified and included MUT 4883 and a *P*. *oceanica* isolate [33]. Within Massarinaceae, MUT 4860 and 4863, which grouped closely to MUT 4887 *Massarina rubi* (a species occurring on at least eight plant families as saprotroph), represent separate entities. Within Sporormiaceae, the strain MUT 4858 (Sporormiaceae sp.), fell between *Westerdykella* and *Preussia* genera. However, the mycelium was sterile and the reference dataset still needs to be improved by more LSU sequences from type species deposited in public collections.

Capnodiales mainly incorporates saprobes, plant and human pathogens, and endophytes, comprising several lichenized species [54]. Here, the phylogenetic analysis was a powerful tool to resolve the majority of the taxa belonging to this class. Interestingly, MUT 4958 and 5396 seem to form a new taxonomic cluster among the Teratosphaeriaceae [55], which represents a taxonomically complex family with many species still to be phylogenetically resolved [38, 54–57] and their geographic distribution and hosts to be better understood [58].

Sordariomycetes encompass 31% of isolated fungal strains and about 30% of the analysed sterile mycelia; this is one of the largest classes in the Ascomycota, which includes endophytes, plants and animal pathogens, and mycoparasites including several obligate marine fungi [2, 59–61]. Marine Sordariomycetes are also known for their ability to synthesize unique bioactive compounds [6]. Similarly to Pleosporales, the phylogenetic analysis underlines the presence of some putative taxa never described before. In detail, MUT 4889 could represent a new species belonging to Niessliaceae, a family of saprotrophic fungi living on leaves or wood, both in terrestrial and marine ecosystems [60, 62]. The isolate MUT 4861 (identified as Microascaceae sp.) fell within the Microascales, a small order of primarily saprobic fungi of soils, also responsible for plant and human diseases [59, 60], but did not cluster with other taxa, hence, it may represent a new fungal entity. Further analyses are required for all the putative new taxa/lineages (sequencing of several genetic markers and culturing on different media) to better understand their taxonomic position and enhance the chance to visualize reproductive structures.

Finally, Eurotiomycetes represents the third most representative class, with 23% of the recovered species. The high frequency of Eurotiomycetes recovery in the present study is concordant with many other marine substrata and sea ecosystems [47, 63, 64]. However, due to their high growth rate and sporulation, their dominance could be overestimated.

*Penicillium* was the most frequently found genus in the present study. This genus is cosmopolitan and shows tolerance to different environmental conditions, such as those shaping different kind of marine habitats. *P*. *antarcticum*, the most widespread species on *F*. *petiolata*, has already been reported in marine waters, sediments and sponges [64–66]. All the other isolated *Penicillium* species have already been reported from seawater, algae, sponges, sands, deep-sediments and/or other abiotic matrices collected from different marine habitats around the world [64–69], confirming *Penicillium* genus as widespread in the marine environment.

*Cladosporium* spp. and *Arthrinium* spp. were also retrieved in both sampling sites. These genera are frequently isolated from terrestrial environments [70, 71] but include species that colonize marine substrata, saline and hypersaline environments [12, 41, 47–49, 72, 73].

Additionally, several taxa recovered in the present study represent new records for marine environment: some of them usually behave as saprobes and are widespread in terrestrial habitats (*i*.*e*. *Acremonium sclerotigenum*, *Cladosporium allicinum*, *Gliomastix masseei*, *Myceliophthora verrucosa*, *Penicillium palitans*) (S1 Table). Other fungal taxa are rare even in terrestrial environments, *i*.*e*. *Knufia petricola* (syn. *Sarcinomyces petricola*), a meristematic-black yeast living on stone as unlichenized fungus [74, 75], *Ramularia eucalypti* (anamorph of *Mycosphaerella thailandica*), a species collected from several locations in Italy causing severe leaf spotting symptoms of *Eucalyptus* trees [57, 58, 76] *Valsonectria pulchella* only know from the type specimen isolated from decaying branches of *Melia azedarach* [77] and *Verrucocladosporium dirinae*, a mycophycobiont isolated from lichen *Dirina massiliensis* [39, 54], and from Italian monumental sites [74].

This work has highlighted the presence of a relevant number of taxa associated to *F*. *petiolata* and contributes significantly to the understanding of new phylogenetic lineages in important fungal classes. Further studies dealing with marine algae as hotspots for marine fungi would be needed. Knowing that many species are refractory to cultivation, an approach blending metagenomics and culturomics would definitely unveil complementary information on *F*. *petiolata-*associated fungi, their ecological roles and functions [78, 79].

Finally, it must be underlined that several strains isolated in this work have been recently shown to be an untapped source of secondary metabolites of biotechnological importance: i) Roussoellaceae sp. 2 (MUT 4859), *Massarina* sp. 1 (MUT 4860), Microascaceae sp. (MUT 4861) *B*. *bassiana* (MUT 4865), *K*. *petricola* (MUT 4979) produce antimicrobial compounds effective against Multi Drug Resistant Bacteria [80]; ii) Roussoellaceae sp. 2 (MUT 4859), *A*. *sclerotigenum* (MUT 4872), *M*. *verrucosa* (MUT 4878), *A*. *salicis* (MUT 4879) secrete novel biosurfactants agents belonging to hydrophobins, class I and II [81]. These biological activities indicate possible relevant ecological roles of algicolous fungi that should be further investigated.

## Conclusions

The green alga *F*. *petiolata* represents a very promising and interesting substrate hosting an uncharted and untapped high fungal diversity. Here, a quali-quantitative analysis of the culturable mycobiota was performed and represents, to the best of our knowledge, the first report of fungi associated to a green alga in the Mediterranean Sea. Several taxa reported in the present study represent new records for the marine environment, for which physiological features and ecological roles have yet to be clarified. Finally, since all the identified strains have been deposited in a public Biological Resource Centre, this work contributes to our understanding of the algal-inhabiting mycobiota and will allow the exploitation of such untapped resources for putative biotechnological applications.

## Supporting information

S1 Dataset List of sequences, with NCBI accession numbers, used to build each phylogenetic tree. (DOCX)

S1 Fig Marine fungal strains isolated from *F*. *petiolata*: (a) MUT 4979 *Knufia petricola* sterile mycelium, hyphae with thick-walled cells; (b) MUT 4860 *Massarina* sp. 1, sterile mycelium with thick-walled cells; (c) MUT 4963 *Rhexocercosporidium carotae* conidia; (d) MUT 4861 Microascaeae sp., conidiogenous cells with immature (sx) and mature conidia (dx); (e) MUT 4958 Teratosphaeriaceae sp. 1, pycnidium with conidia; (f) MUT 5053 *Sedecimiella taiwanensis*, hyphae, conidiogenous cells and conidia; (g) MUT 4941 *Pyrenochaetopsis* sp., pycnidia; (h) MUT 4858 Sporormiaceae sp., pycnidia with conidia (sx), immature conidial chains and mature conidiogenous cells with attached conidia (dx); (i) MUT 4886 Roussoellaceae sp. 3, pycnidium with conidia; (j) MUT 4890 *Valsonectria pulchella*, conidiophores with phialides (sx), phialides with conidia (center), detail of the phialid-conidiogenous cells (dx); (k) MUT 4863 *Massarina* sp. 2, colony on different media after three weeks. Scale bars (a-j): 20 μm.
(TIF)

S2 Fig Non-Metric Multi Dimensional Scaling (NMDS) analysis performed on the taxa associated to each thallus per site. 1–10 algal thalli from Ghiaie (green); 11–20 algal thalli from Margidore (red). The main group is highlited in the inset.
(PNG)

S1 Table Marine fungal entities isolated from *F*. *petiolata* and recovered in other marine substrates and environments. (DOCX)

S2 Table PCR amplification program details. (DOCX)

S3 Table Presence/absence matrix of the taxa retrieved in 10 thalli of *F*. *petiolata* per each site analysed. (DOCX)
