# Supplementary material for: The culturable mycobiota of Flabellia petiolata: First survey of marine fungi associated to a Mediterranean green alga
Source: PLoS One. 2017 Apr 20;12(4):e0175941. doi: 10.1371/journal.pone.0175941 (PMC5398637; doi:10.1371/journal.pone.0175941)
Supplement: S1 Dataset — (DOCX) [file pone.0175941.s001.docx]

**S1 Dataset. List of sequences, with NCBI accession number, used to build each phylogenetic tree.**

**CAPNODIALES**

AY281094 Ophiostoma piliferum

DQ008146 Cladosporium cladosporioides CBS 109501

DQ008147 Cladosporium uredinicola CBS 306.84

DQ008148 Davidiella macrospora CBS 138.40

DQ008149 Davidiella tassiana CBS 813.71

DQ247802 Dothidea insculpta CBS 189.58

DQ368627 Ophiostoma ulmi CBS 298.87

DQ470984 Dothiora cannabinae

DQ678091 Cercospora beticola

EU040237 Rachicladosporium luculiae

EU040244 Verrucocladosporium dirinae CBS 112794

EU436763 Devriesia strelitziae CBS 122379

EU707865 Teratosphaeria knoxdaviesii CBS 122898

FJ790305 Toxicocladosporium rubrigenum CBS 124158

FJ267700 Catenulostroma abietis

GQ852597 Mycosphaerella pini CBS 112498

GQ852634 Pseudocercospora paraguayensis CBS 111317

FJ493208 Phaeophleospora eugeniicola

FJ493209 Phaeophleospora eugeniicola

EU040243 Toxicocladosporium irritans CBS 185.58

EU167598 Mycosphaerella stromatosa CBS 101953

EU019246 Batcheloromyces leucadendri CBS 110892

EU019247 Batcheloromyces proteae CBS 110696

EU019251 Catenulostroma chromoblastomycosum CBS 597.97

EU019253 Catenulostroma germanicum CBS 539.88

EU019255 Catenulostroma microsporum CBS 110890

EU019263 Cladosporium bruhnei CBS 188.54

EU019267 Uwebraunia commune CBS 114238

EU019284 Ramularia pratensis var. pratensis

EU019285 Ramularia sp. CBS 324.87

EU019295 Teratosphaeria stellenboschiana

EU019303 Teratosphaeria aff. nubilosa CBS 114419

EU019306 Teratosphaeria secundaria CBS 115608

EU009458 Graphiopsis chlorocephala

AY490776 Mycosphaerella punctiformis CBS 113265

GU214400 Capnodium coffeae CBS 147.52

GU214409 Cladosporium cladosporioides

GU214410 Davidiella tassiana CBS 723.79

GU214414 Devriesia hilliana

GU214415 Devriesia lagerstroemiae

GU214416 Devriesia staurophora

GU214419 Dissoconium aciculare CBS 204.89

GU214435 Mycosphaerella endophytica

GU214449 Mycosphaerella sp. CBS 111166

GU214450 Mycosphaerella sp. CBS 111167

GU214470 Pseudocercospora angolensis

GU214472 Pseudocercospora cordiana

GU214475 Pseudocercospora griseola f. griseola CBS 194.47

GU214483 Pseudocercospora vitis

GU214484 Rachicladosporium cboliae

GU214489 Teratosphaeria molleriana

GU214505 Teratosphaeria cryptica

GU214654 Cercospora capsici strain CPC 12307

GU214663 Lecanosticta acicola CBS 871.95

GU214673 Pseudocercospora cruenta

GU214676 Pseudocercospora humuli

GU214677 Pseudocercospora kaki

GU214680 Pseudocercospora pallida

GU214687 Ramichloridium apiculatum

GU214691 Mycosphaerella fragariae

GU214692 Ramularia coleosporii

GU214694 Ramularia uredinicola

GU301810 Devriesia strelitziae CBS 122379

GU301831 Leptoxyphium fumago CBS 123.26

NG 027594 Stylodothis puccinioides CBS 193.58

HQ599606 Devriesia xanthorrhoeae CBS128219

GU214675 Pseudocercospora fuligena

JF499860 Devriesia tardicrescens CBS 128770

JF951165 Rachicladosporium pini

JF951168 Devriesia queenslandica

JN712568 Teratosphaeria capensis

JN712569 Teratosphaeria capensis

JN832601 Scorias spongiosa

JQ622104 Uwebraunia dekkeri

JX069859 Devriesia agapanthi

JX915750 Devriesia imbrexigena

JX915749 Devriesia imbrexigena

JQ732980 Mycosphaerella marksii

JQ732985 Cladosporium delicatulum

KC005797 Devriesia shakazului

KC005799 Devriesia stirlingiae

KJ395498 Ramularia endophylla MUT 4389

**LEOTIOMYCETES**

NG 027608 Monilinia laxa CBS 122031

NG 027597 Lachnum virgineum

KJ663873 Bulgaria inquinans CBS 315.71

KF836623 Leotia lubrica

KF636784 Helotiaceae sp. MUT 4416

KF636779 Helotiales sp. MUT 4402

KF636778 Helotiales sp. MUT 4401

KF636777 Helotiales sp. MUT 4412

KF636776 Helotiales sp. MUT 4411

KF053587 Cadophora luteo-olivacea

KF053584 Cadophora malorum

KF05358 Cadophora luteo-olivacea

KC834020 Arbusculina fragmentans

KC833185 Cudonia sp.

JQ768393 Crocicreas sp.

JN938890 Botrytis aclada

JN938889 Botryotinia fuckeliana

JN673046 Hymenoscyphus fructigenus

HM140552 Lophodermium pinastri

HM116760 Cadophora luteo-olivacea

EU998917 Articulospora tetracladia

EU940088 Cyathicula microspora

EU883430 Tetracladium maxilliforme

EU883428 Tetracladium furcatum

EU883419 Tetracladium apiense

EU107209 Cyttaria darwinii

DQ470954 Meria laricis

DQ470944 Cudoniella clavus

DQ470942 Mollisia cinerea

DQ257365 Neobulgaria pura

DQ257356 Holwaya mucida

DQ257349 Chlorociboria sp.

DQ247801 Dermea acerina CBS 161.38 2

DQ227262 Hyphodiscus hymeniophilus CBS 687.74

DQ227260 Hyphodiscus hymeniophilus CBS 231.75

AY789431 Hymenoscyphus scutula

AY789423 Mitrula paludosa

AY789412 Hydrocina chaetocladia

AY789409 Sarcoleotia globosa

AY789407 Scleromitrula shiraiana

AY789404 Mitrula borealis

AY789402 Vibrissea truncorum

AY789397 Microglossum olivaceum

AY789391 Peziza varia

AY789383 Vibrissea albofusca

AY789373 Cudoniella clavus

AY789365 Ombrophila violacea

AY789347 Sclerotinia sclerotiorum strain

AY789344 Bulgaria inquinans

AY789313 Trichoglossum hirsutum

AY640965 Sarea resinae

AY544683 Monilinia fructicola

AY544674 Lachnum cf. bicolor

AY544662 Neofabraea malicorticis

AY544651 Botryotinia fuckeliana

AY261178 Orbilia delicatula

AY261147 Arthrobotrys oligospora

AY004334 Lophodermium pinastri

AJ406399 Ascocoryne sarcoides

AF433142 Spathularia flavida

AF356694 Fabrella tsugae

AB496937 Mycochaetophora gentianae

AB469688 Rhexocercosporidium carotae

AB469686 Mycochaetophora gentianae

AB469680 Mycochaetophora sp.

AB022393 Podosphaera tridactyla var. tridactyla

AB022364 Sawadaea polyfida var. japonica

AB022362 Blumeria graminis f. sp. br

**PLEOSPORALES**

AY004343 Westerdykella cylindrica

AY016357 Byssothecium circinans

AY016369 Trematosphaeria heterospora

AY293792 Didymella cucurbitacearum

AY544645 Cochliobolus heterostrophus

AY544672 Pyrenophora tritici-repentis

AY544684 Phaeosphaeria avenaria

AY607730 Arthopyrenia salicis

DQ384108 Ulospora bilgramii CBS 110020

DQ470946 Leptosphaeria maculans

GQ203753 Westerdykella dispersa CBS 297.56

DQ499596 Pyrenophora phaeocomes

DQ678045 Cochliobolus sativus

DQ678049 Pleospora herbarum

DQ678056 Preussia minima

DQ678063 Phaeosphaeria eustoma

DQ678066 Phoma herbarum

DQ678069 Lophiostoma crenatum

DQ678071 Herpotrichia diffusa

DQ678072 Trematosphaeria pertusa

DQ678080 Herpotrichia juniperi

DQ678082 Alternaria alternata

DQ678086 Montagnula opulenta

DQ678096 Pyrenochaeta nobilis

DQ782384 Lophiostoma arundinis

AB369267 Lophiostoma sagittiforme

EU754186 Phoma herbarum CBS 615.75

EU754137 Didymella pisi CBS 126.54

EU754139 Paraconiothyrium tiliae CBS 265.94

EU754167 Leptosphaerulina australis CBS 939.69

EU754174 Paraphaeosphaeria minitans strain CBS 122786

EU754175 Parastagonospora nodorum strain CBS 110109

EU754190 Paraphoma radicina strain CBS 102875

EU754197 Paraconiothyrium fuscomaculans strain CBS 116.16

EU754198 Pyrenochaeta cava CBS 115979

AB469680 Mycochaetophora sp.

FJ515635 Didymella vitalbina CBS 123706

AB496937 Mycochaetophora gentianae

GQ387591 Phaeosphaeria oryzae CBS 110110

AB521743 Tingoldiago graminicola

GQ925844 Platystomum scabridisporum

GQ925848 Biatriospora marina

GQ925851 Massarina velataspora

GQ925853 Massarina ramunculicola

AB524597 Lophiotrema neoarundinaria

AB524621 Roussoella hysterioides

AB524622 Roussoella hysterioides

AB524623 Roussoella pustulans

AB524624 Roussoella intermedia

AB524625 Roussoellopsis tosaensis

GU301793 Aigialus grandis

GU301809 Corynespora olivacea CBS 114450

GU301826 Leptosphaeria biglobosa CBS 303.51

GU301840 Massarina eburnea CBS 473.64

GU301872 Preussia lignicola CBS 264.69

GQ203720 Westerdykella angulata

GQ203725 Preussia terricola CBS 317.65

GU456317 Alternaria maritima CBS 126.60

GU456328 Phaeosphaeria juncophila CBS 575.86

GU479776 Aigialus mangrovis

GU479778 Aigialus parvus

GU479784 Caryosporella rhizophorae

GU479793 Massarina ricifera

GU479802 Verruculina enalia

GU479807 Phaeosphaeria olivacea

GU479808 Phaeosphaeria spartinicola

GU238054 Westerdykella capitulum CBS 337.65

GU238184 Stagonosporopsis dorenboschii CBS 320.90

GU238187 Stagonosporopsis heliopsidis

GU238192 Stagonosporopsis loticola CBS 562.81

GQ254665 Massarina corticola

GQ387590 Phaeosphaeria caricicola CBS 603.86

GQ387603 Pyrenochaeta acicola CBS 101634

GQ387605 Cucurbitaria berberidis CBS 394.84

GQ387609 Pyrenochaeta corni CBS 102828

GQ387612 Pyrenochaeta lycopersici CBS 267.59

GQ387614 Biatriospora mackinnonii CBS 110022

GQ387615 Pyrenochaeta nobilis CBS 292.74

GQ387620 Pyrenochaeta quercina CBS 297.74

GQ387623 Pyrenochaeta unguis-hominis CBS 111112

GQ387625 Pyrenochaetopsis decipiens CBS 165.89

GQ387626 Pyrenochaetopsis indica CBS 124454

GQ387627 Pyrenochaetopsis leptospora CBS 101635

GQ387630 Pyrenochaetopsis microspora CBS 101333

FJ161174 Hysterobrevium smilacis CBS 114601

FJ161178 Psiloglonium simulans CBS 206.34

FJ161180 Hysterium angustatum CBS 236.34

AB553749 Prosthemium orientale

AB553765 Prosthemium canba

AB619025 Lophiotrema vagabundum

Pleospora typhicola CBS 132.69

JF740327 Pleurophoma pleurospora CBS 130329

GQ387584 Paraphoma fimeti CBS 170.70

JQ318010 Phoma foliaceiphila

JQ238625 Phoma cladoniicola CBS 128025

JQ238643 Phoma caloplacae CBS 129338

JX442037 Bambusicola massarinia

KF251662 Leptosphaeria albopunctata CBS 254.64

KF251670 Paraphoma chrysanthemicola CBS 522.66

KF636771 Didymellaceae sp. MUT 4313

KF636772 Massarina rubi MUT 4323

KF636773 Pyrenochaeta sp. MUT 4378

KF636774 Pleospora typhicola MUT 4379

KF636775 Roussoellaceae sp. MUT 4397

KF636780 Cucurbitariaceae sp. MUT 4403

KF636781 Phaeosphaeriaceae sp. MUT 4404

KF636782 Massarina sp. 4405

KF636783 Biatriospora sp. MUT 4407

KF636785 Pleosporales sp. MUT 4417

KF636786 Roussoellaceae sp. MUT

KF636787 Lentitheciaceae sp. 4419

JX681090 Keissleriella cladophila CBS 104.55

KJ474839 Neoroussoella bambusae

KJ395496 Pleosporales sp. MUT 4273

KJ395497 Pyrenochaeta acicola MUT 4382

**SORDARIOMYCETES**

U17404 Melanospora fallax

U17405 Melanospora zamiae

U17416 Pseudonectria rousseliana

U17425 Verticillium dahliae

U46882 Aniptodera chesapeakensis

U46885 Halosphaeria appendiculata

U46886 Halosarpheia fibrosa

U46890 Lignincola laevis

U47825 Chaetomium globosum

U47826 Claviceps paspali

U47823 Cercophora septentrionalis

U47824 Ceratocystis virescens

U47828 Daldinia concentrica

U47829 Diatrype disciformis

U47830 Diaporthe phaseolorum

U47833 Hypocrea schweinitzii

U47835 Microascus trigonosporus

U47841 Xylaria hypoxylon

AF064641 Chaetosphaeria ovoidea

AF064643 Lasiosphaeria ovina

AF132330 Sordaria fimicola

AF195641 Lulworthia sp. JK4843

AF221009 Ceratocystis fimbriata

AF193237 Nectria cinnabarina

AF160230 Hypomyces aurantius

AF160241 Hypomyces orthosporus

AF213027 Hypomyces chlorinigenus

AF279410 Seynesia erumpens

AF327374 Cordyceps militaris

AF286410 Farrowia seminuda

AF286411 Neurospora crassa

AF382357 Pestalotiopsis versicolor

AF382367 Bartalinia bischofiae

AF382377 Seiridium cardinale

AF382380 Discostroma sp.

AY015635 Valetoniellopsis laxa

AF339520 Beauveria caledonica

AF362557 Gaeumannomyces graminis var. graminis

AF362568 Discula destructiva

AF452029 Amphisphaeria umbrina

AF452030 Monographella nivalis

AF452038 Arecophila bambusae

AF431950 Cephalotheca sulfurea CBS 135.34

AF510497 Trichoderma reesei

AF491260 Corollospora maritima

AF491270 Lulworthia fucicola

AF408343 Cryptodiaporthe corni CBS245.90

AF408387 Valsa ceratosperma

AY083821 Camarops microspora

AY083826 Cryptosphaeria eunomia var. eunomia

AY083827 Graphostroma platystoma

AY083829 Hypoxylon fragiforme

AY083830 Clypeosphaeria uniseptata

AY083834 Hyponectria buxi

AF543792 Aphysiostroma stercorarium

AF543793 Hypomyces polyporinus

AB067706 Cordyceps crassispora

AB067709 Cordyceps sinensis

AB067710 Cordyceps sinensis

AF539473 Neptunella longirostris

AF539476 Nais inornata

AJ459309 Hypomyces subiculosus

AY281094 Ophiostoma piliferum

AY346265 Camarops petersii

AY346266 Camarops tubulina

AY346267 Camarops ustulinoides

AY346276 Coniochaetidium savoryi

AY346292 Melanochaeta hemipsila

AY346295 Ophioceras tenuisporum

AY346297 Coniochaeta discoidea

AY346299 Pseudohalonectria lignicola

AY346300 Schizothecium curvisporum

AY346301 Sordaria macrospora

AY346304 Valsonectria pulchella

AY346305 Zopfiella ebriosa CBS 111.75

AY545726 Hydropisphaera erubescens

AY545729 Chaetomium globosum

AY489709 Melanopsamma pomiformis

AY489718 Niesslia exilis CBS357.70

AY489720 Niesslia exilis CBS560.74

AY489725 Stilbocrea macrostoma

AY489728 Clonostachys pityrodes

AY489731 Myrothecium inundatum

AY489734 Cosmospora coccinea

AY489737 Verticillium dahliae

AJ583484 Hypomyces siamensis

AY780050 Anthostomella sp.

AY780053 Bombardia bombarda

AY780054 Camarops amorpha

AY780075 Podospora fimbriata CBS 144.54

AY886545 Ascopolyporus philodendrus

AF382386 Pleospora herbarum CBS 191.86

AY999113 Cercophora caudata CBS 606.72

DQ368625 Microascales sp. CBS 126.78

DQ368626 Gnomonia ribicola

DQ368627 Ophiostoma ulmi CBS 298.87

DQ368628 Chaetomium elatum

DQ368629 Discostroma botan

DQ368630 Apiospora bambusae

DQ368631 Apiospora setosa

DQ414530 Apiospora montagnei

DQ471018 Apiospora montagnei

DQ470969 Petriella setifera

DQ470980 Gelasinospora tetrasperma

DQ522856 Lulworthia grandispora

DQ836907 Doratomyces stemonitis

DQ414531 Seiridium papillatum CBS 340.97

DQ414532 Seiridium unicorne CBS 908.85

DQ414533 Seiridium eucalypti CBS 343.97

DQ862027 Bionectria ochroleuca

EF591763 Trichoderma atroviride

EU334676 Beauveria bassiana

EU334679 Beauveria bassiana

AM779860 Hypomyces australis

FJ360521 Neurospora crassa

FJ345358 Scedosporium apiospermum

AM909688 Thielavia subthermophila

FJ666351 Chaetomidium fimeti CBS 114382

FJ666352 Chaetomidium trichorobustum CBS 563.67

FJ666353 Chaetomidium leptoderma CBS 538.74

FJ666356 Chaetomidium pilosum CBS 335.67

FJ666357 Chaetomidium subfimeti CBS 169.71

FJ666358 Chaetomidium fimeti CBS 168.71

FJ666360 Chaetomidium galaicum

FJ666361 Chaetomidium galaicum CBS 113678

FJ666364 Corynascus sepedonium

FJ666365 Chaetomium longicolleum

HM135162 Cordyceps militaris

HM635077 Chamaeleomyces granulomatis

HM119590 Cordyceps gunnii

GU180648 Gibellulopsis nigrescens

HM448442 Thielavia subthermophila CBS 125981

HM595578 Chaunopycnis sp.

HM595579 Chaunopycnis alba

HM595581 Coniochaeta sp.

JF429894 Beauveria bassiana

JN411084 Cordyceps taishanensis

JQ067896 Myceliophthora fergusii

JQ067899 Thielavia australiensis

JQ067900 Myceliophthora hinnulea

JQ067901 Remersonia thermophila

JQ067902 Melanocarpus albomyces

JN990695 Chamaeleomyces granulomatis strain

JQ780662 Pochonia bulbillosa isolate

JX280761 Thielavia terrestris CBS 492.74

KC987242 Acremonium sclerotigenum

KJ443143 Acrostalagmus luteoalbus

KJ443144 Acrostalagmus luteoalbus

LK932704 Myceliophthora verrucosa

LK932705 Myceliophthora verrucosa

KT207704 Gibellulopsis nigrescens

HM451496 Sedecimiella taiwanensis
