# Supplementary figures and images for: The culturable mycobiota of Flabellia petiolata: First survey of marine fungi associated to a Mediterranean green alga
Source: PLoS One. 2017 Apr 20;12(4):e0175941. doi: 10.1371/journal.pone.0175941 (PMC5398637; doi:10.1371/journal.pone.0175941)

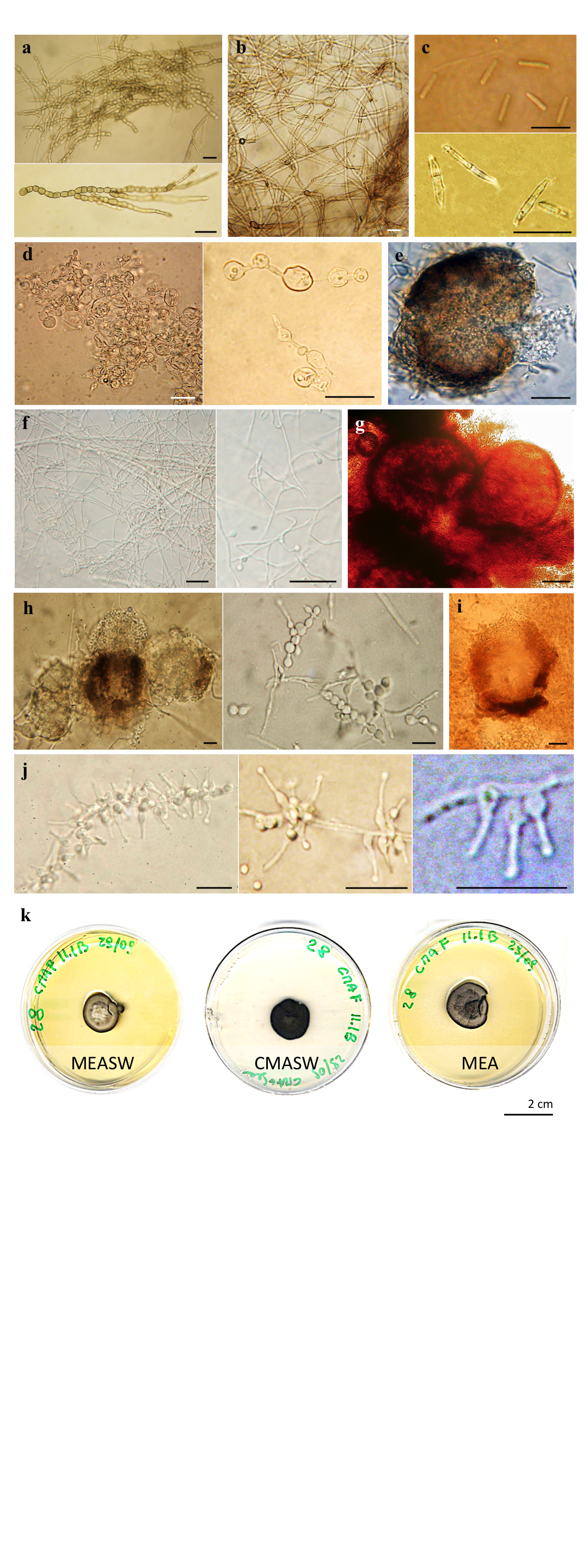

Supplement: S1 Fig — Scale bars (a-j): 20 μm. (TIF) [file pone.0175941.s002.tif]

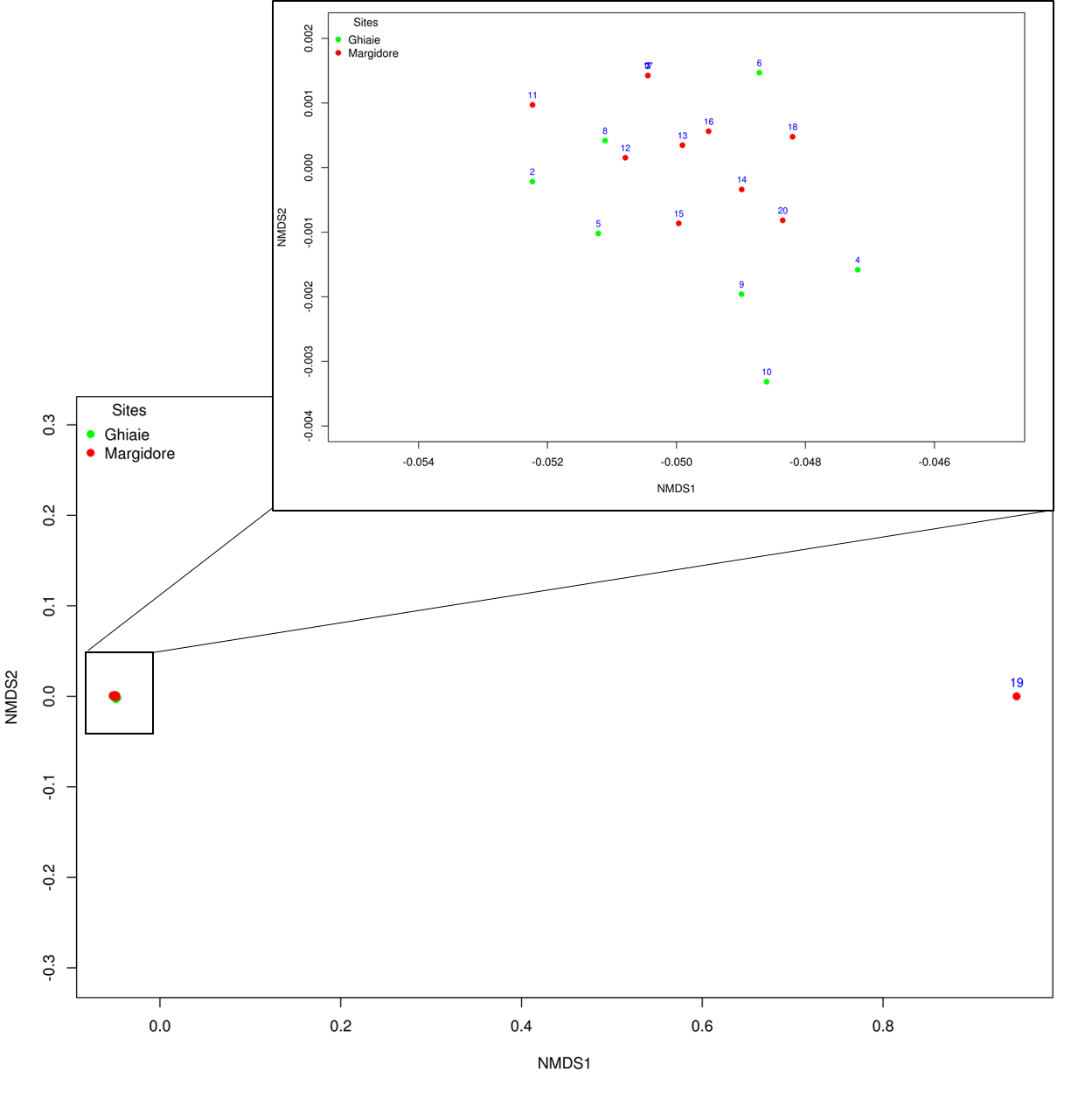

Supplement: S2 Fig — 1–10 algal thalli from Ghiaie (green); 11–20 algal thalli from Margidore (red). The main group is highlited in the inset. (PNG) [file pone.0175941.s003.png]
