# Supplementary material for: The culturable mycobiota of Flabellia petiolata: First survey of marine fungi associated to a Mediterranean green alga
Source: PLoS One. 2017 Apr 20;12(4):e0175941. doi: 10.1371/journal.pone.0175941 (PMC5398637; doi:10.1371/journal.pone.0175941)
Supplement: S1 Table — (DOCX) [file pone.0175941.s004.docx]

**S1 Table. Marine fungal entities isolated from *F. petiolata* and recovered in other marine substrates and environments.**

| **Taxa** | **From MED** | **From ME** | **From algae** |
| --- | --- | --- | --- |
| ***Acremonium breve*** (Sukapure & Thirum.) W. Gams |  | [S1] [S2] [S3] [S4] |  |
| ***Acremonium sclerotigenum*** (Moreau & R. Moreau ex Valenta) W. Gams |  |  | FR |
| ***Acremonium tumulicola*** Kiyuna, An, Kigawa & Sugiyama |  |  | FR |
| ***Acrostalagmus luteoalbus*** Gams & Schroers |  | [S5] [S6] [S7] |  |
| ***Alternaria alternata*** (Fr.) Keissl. | [S9] [S10] | [S4] [S5] [S6] [S8] [S9] [S10] [S11] [S12] [S13] | *Laurencia* spp. |
| ***Apiospora montagnei*** Sacc. | [S9] | [S5] [S6] [S7] [S9] [S13] | *Sargassum* sp. |
| ***Arthopyrenia salicis*** A. Massal. |  |  | FR |
| ***Arthrinium marii*** Larrondo & Calvo |  |  | FR |
| ***Arthrinium phaeospermum*** (Corda) Ellis | [S9] | [S7] [S9] |  |
| ***Aureobasidium pullulans*** (de Bary) Arna. |  | [S4] [S5] [S7] |  |
| ***Beauveria bassiana*** (Bals.-Criv.) Vuill. | [S9] | [S5] [S9] [S14] [S15] |  |
| ***Biatriospora* sp.** | / | / | / |
| ***Botrytis cinerea*** Pers. | [S8] | [S6] [S8] |  |
| ***Chaetomium globosum*** Kunze |  | [S5] [S6] [S7] [S16] [S17] [S18] | *Ulva pertusa* (**+)***, Polysiphonia urceolata* |
| ***Cladosporium allicinum*** Bensch, & Crous |  |  | FR |
| ***Cladosporium cladosporioides*** (Fresen)V. | [S9] [S11] [S19] | [S5] [S9] [S11] [S14] [S19] |  |
| ***Cladosporium herbarum*** (Pers.) Link | [S9] | [S4] [S5] [S6] [S7] [S9] [S20] |  |
| ***Cladosporium sphaerospermum*** Penz. | [S9] | [S4] [S5] [S9] [S14] |  |
| ***Coprinellus*** **sp.** | / | / | / |
| ***Devriesia* sp.** | / | / | / |
| ***Emericellopsis minima*** Stolk |  | [S21] [S22] |  |
| ***Gibellulopsis nigrescens*** (Pethybr.) Zare, W. Gams & Summerb. | [S9] | [S9] [S13] [S23] | *Sargassum sp.* |
| ***Gliomastix masseei*** (Sacc. & Trotter) Matsush. |  |  | FR |
| **Herpotrichiellaceae sp.** | / | / | / |
| **Hypocreales sp.** | / | / | / |
| ***Knufia petricola*** (U. Wollenzien & de Hoog) Gorbushina & Gueidan |  |  | FR |
| ***Massarina rubi*** (Fuckel) Sacc. | [S24] | [S24] |  |
| ***Massarina* sp. 1** | / | / | / |
| ***Massarina* sp. 2** | / | / | / |
| **Microascaceae sp.** | / | / | / |
| ***Microascus cirrosus*** Curzi |  | [S5] [S6] |  |
| ***Microascus trigonosporus*** C.W. Emmons & B.O. Dodge |  | [S5] |  |
| ***Myceliophthora verrucosa*** (Stchigel, Cano & Guarro) van den Brink & Samson |  |  | FR |
| ***Penicillium antarcticum*** Hocking & McRae |  | [S25] |  |
| ***Penicillium atramentosum*** Thom |  | [S26] [S27] |  |
| ***Penicillium brevicompactum*** Dierckx | [S8] [S9] [S11] [S28] | [S5] [S8] [S9] [S11] [S14] [S27] [S28] [S29] | *Cystoseira* sp. |
| ***Penicillium chrysogenum*** Thom | [S8] [S9] [S28] [S30] | [S4] [S5] [S6] [S7] [S8] [S9] [S14] [S27] [S28] [S30] [S31] [S32] [S33] | *Laurencia* sp., *Hypnea* complex, |
| ***Penicillium commune*** Thom |  | [S4] [S5] [S34] [S35] [S36] [S37] [S38] |  |
| ***Penicillium crustosum*** Thom | [S28] | [S27] [S28] [S39] [S40] [S41] |  |
| ***Penicillium expansum*** Link | [S9] | [S5] [S9] [S11] [S14] [S27] [S42] [S43] |  |
| ***Penicillium palitans*** Westling |  |  | FR |
| ***Penicillium simplicissimum*** (Oudem.) Thom |  | [S5] [S14] |  |
| ***Penicillium solitum*** Westling |  | [S5] [S14] [S27] [S44] |  |
| ***Penicillium* sp.** | / | / | / |
| ***Peniophora* sp.** | / | / | / |
| ***Pyrenochaetopsis* sp.** | / | / | / |
| ***Ramularia eucalypti*** Crous |  | [S45] |  |
| ***Rhexocercosporidium carotae*** (Årsvoll) U. Braun | [S26] | [S26] |  |
| **Roussoellaceae sp**. **1** | / | / | / |
| **Roussoellaceae sp**. **2** | / | / | / |
| **Roussoellaceae sp**. **3** | / | / | / |
| **Roussoellaceae sp. 4** | / | / | / |
| **Roussoellaceae sp. 5** | / | / | / |
| **Roussoellaceae sp. 6** | / | / | / |
| ***Sarocladium strictum*** (W. Gams) Summerb. |  | [S5] |  |
| ***Schizophyllum commune*** Fr. | [S9] | [S5] [S9] |  |
| ***Sedecimiella taiwanensis*** K.L. Pang, Alias & E.B.G. Jones |  | [S5] [S46] [S47] |  |
| **Sporormiaceae sp.** | / | / | / |
| ***Talaromyces*** ***variabilis*** (Sopp) Samson, Yilmaz, Frisvad & Seifert |  | [S6] |  |
| **Teratosphaeriaceae sp. 1** | / | / | / |
| **Teratosphaeriaceae sp. 2** | / | / | / |
| **Trichomeriaceae sp.** | / | / | / |
| ***Valsonectria pulchella*** Speg. |  |  | FR |
| ***Verrucocladosporium dirinae*** K. Schub., Aptroot & Crous |  |  | FR |

MED = recorded from Mediterranean Sea; ME = recorded from marine environments; FR = first record from *F. petiolata* (this study) and (**+)** = from other green algae.

[S1] Borzykh OG, Zvereva LV, Comparison of fungal complexes of Japanese scallop *Mizuhopecten yessoensis* (Jay, 1856) from different areas in the Peter the Great Bay of the Sea of Japan. Microbiology, 2014; 83:684-9.

[S2] Kirichuk NN, Pivkin MV, Polokhin OV, Fungal assemblages of submarine soils of the eastern Sakhalin shelf. Russ J Mar Biol, 2012;38:375-80.

[S3] Steiman R, Guiraud P, Sage L, Seigle-Murandi F, Soil mycoflora from the Dead sea oases of Ein Gedi and Einot Zuqim (Israel). Anton Leeuw I J G, 1997;72,261-70.

[S4] Kis-Papo T, Grishkan I, Oren A, Wasser SP, Nevo E, Spatiotemporal diversity of filamentous fungi in the hypersaline Dead Sea. Mycol Res, 2001;105:749-56.

[S5] Jones EG, Suetrong S, Sakayaroj J, Bahkali AH, Abdel-Wahab MA, Boekhout T, Pang KL, Classification of marine Ascomycota, Basidiomycota, Blastocladiomycota and Chytridiomycota. Fungal Divers, 2015;73:1-72.

[S6] Clipson N, Landy E, Otte M, Fungi, in: Costello MJ et al. (Eds.) European register of marine species: a check-list of the marine species in Europe and a bibliography of guides to their identification. Collection Patrimoines Naturels, 2001;50: pp. 15-19.

[S7] Landy ET, Jones GM, What is the fungal diversity of marine ecosystems in Europe? Mycologist, 2006;20:15-21.

[S8] López-Legentil S, Erwin PM, Turon M, Yarden O, Diversity of fungi isolated from three temperate ascidians. Symbiosis, 2015;66:99-106.

[S9] Panno L, Bruno M, Voyron S, Anastasi A, Gnavi G, Miserere L, Varese GC, Diversity, ecological role and potential biotechnological applications of marine fungi associated to the seagrass *Posidonia oceanica*. New Biotechnol, 2013:30:685-6.

[S10] Shaaban M, Shaaban KA, Abdel-Aziz MS, Seven naphtho-γ-pyrones from the marine-derived fungus *Alternaria alternata*: structure elucidation and biological properties. Organic and medicinal chemistry letters, 2012;2:1.

[S11] Atalla MM, Zeinab HK, Eman RH, Amani AY, Abeer AAEA, Screening of some marine-derived fungal isolates for lignin degrading enzymes (LDEs) production. Agriculture and Biology Journal of North America, 2010;1:591-9.

[S12] Gao SS, Li XM, Wang BG, Perylene derivatives produced by *Alternaria alternata*, an endophytic fungus isolated from *Laurencia* species. Nat Prod Commun, 2009;4:1477-80.

[S13] Hong JH, Jang S, Heo YM, Min M, Lee H, Lee YM, Lee H, Kim JJ, Investigation of marine-derived fungal diversity and their exploitable biological activities. Mar Drugs, 2015;13:4137-55.

[S14] Kirichuk NN, Pivkin, MV, Filamentous fungi associated with the seagrass *Zostera marina* Linnaeus, 1753 of Rifovaya Bay (Peter the Great Bay, the Sea of Japan). Russ J Mar Biol, 2015;41:351-5.

[S15] Yamazaki H, Rotinsulu H, Kaneko T, Murakami K, Fujiwara H, Ukai K, Namikoshi M, A new dibenz [b, e] oxepine derivative, 1-hydroxy-10-methoxy-dibenz [b, e] oxepin-6, 11-dione, from a marine-derived fungus, *Beauveria bassiana* TPU942. Mar Drugs, 2012;10,2691-7.

[S16] Kis-Papo T, Oren A, Wasser SP, Nevo E, Survival of filamentous fungi in hypersaline Dead Sea water. Microb Ecol, 2003;45,183-90.

[S17] Debbab A, Aly AH, Proksch P, Bioactive secondary metabolites from endophytes and associated marine derived fungi. Fungal Divers, 2011;49:1-12.

[S18] Wang S, Li XM, Teuscher F, Li DL, Diesel A, Ebel R et al. Chaetopyranin, a benzaldehyde derivative, and other related metabolites from *Chaetomium globosum*, an endophytic fungus derived from the marine red alga *Polysiphonia urceolata*. J Nat Prod, 2006;69:1622-5.

[S19] Garzoli L, Gnavi G, Varese GC, Picco AM, Mycobiota associated with the rhodophyte alien species *Asparagopsis taxiformis* (Delile) Trevisan de Saint‐Léon in the Mediterranean Sea. Mar Ecol, 2015;36:959-68.

[S20] Jadulco R, Brauers G, Edrada RA, Ebel R, Wray V, Proksch, P, New metabolites from sponge-derived fungi *Curvularia lunata* and *Cladosporium herbarum*. J Nat Prod, 2002;65:730-3.

[S21] Pinheiro Â, Dethoup T, Bessa J, Silva AM, Kijjoa A, A new bicyclic sesquiterpene from the marine sponge associated fungus *Emericellopsis minima*. Phytochem Lett, 2012;5:68-70.

[S22] Höller U, Wright AD, Matthee GF, Konig GM, Draeger S, Hans-Jürgen AUST, Schulz B, Fungi from marine sponges: diversity, biological activity and secondary metabolites. Mycol Res, 2000;104;1354-65.

[S23] Garzoli L, Gnavi G, Tamma F, Tosi S, Varese GC, Picco AM, Sink or swim: updated knowledge on marine fungi associated with wood substrates in the Mediterranean Sea and hints about their potential to remediate hydrocarbons. Prog Oceanogr, 2015;137:140-8.

[S24] Gnavi G, Ercole E, Panno L, Vizzini A, Varese GC, Dothideomycetes and Leotiomycetes sterile mycelia isolated from the Italian seagrass *Posidonia oceanica* based on rDNA data. SpringerPlus, 2014;3:1.

[S25] Park MS, Lee EJ, Fong JJ, Sohn JH, Lim YW, A new record of *Penicillium antarcticum* from marine environments in Korea. Mycobiology, 2014;42:109-13.

[S26] Wang L, Zou S, Yin S, Liu H, Yu W, Gong Q, Construction of an effective screening system for detection of *Pseudomonas aeruginosa* quorum sensing inhibitors and its application in bioautographic thin-layer chromatography. Biotechnol lett, 2011;33:1381-7.

[S27] Park MS, Fong JJ, Oh SY, Kwon KK, Sohn JH, Lim YW, Marine-derived *Penicillium* in Korea: diversity, enzyme activity, and antifungal properties. Anton Leeuw Int J G, 2014;106:331-45.

[S28] Paz Z, Komon-Zelazowska M, Druzhinina IS, Aveskamp MM, Shnaiderman A, Aluma Y et al. Diversity and potential antifungal properties of fungi associated with a Mediterranean sponge. Fungal Divers, 2010;42:17-26.

[S29] Rovirosa J, Diaz-Marrero ANA, Darías J, Painemal K, San Martín A, Secondary metabolites from marine *Penicillium brevicompactum*. J Chil Chem Soc, 2006;51:775-8.

[S30] Bringmann G, Lang G, Gulder TA, Tsuruta H, Mühlbacher J, Maksimenka K et al. The first sorbicillinoid alkaloids, the antileukemic sorbicillactones A and B, from a sponge-derived *Penicillium chrysogenum* strain. Tetrahedron, 2005;61, 7252-65.

[S31] Zhu HY, Tian Y, Hou YH, Wang TH, Purification and characterization of the cold-active alkaline protease from marine cold-adaptive *Penicillium chrysogenum* FS010. Mol Biol Rep, 2009;36:2169-74.

[S32] Gao SS, Li XM, Du FY, Li CS, Proksch P, Wang BG, Secondary metabolites from a marine-derived endophytic fungus *Penicillium chrysogenum* QEN-24S. Mar Drugs, 2010;9:59-70.

[S33] Yang G, Yun K, Nenkep VN, Choi HD, Kang JS, Son BW, Induced production of halogenated diphenyl ethers from the marine‐derived fungus *Penicillium chrysogenum*. Chem Biodivers, 2010;7:2766-70.

[S34] Gao SS, Li XM, Zhang Y, Li CS, Cui CM, Wang BG, Comazaphilones A- F, azaphilone derivatives from the marine sediment-derived fungus *Penicillium commune* QSD-17. J Nat Prod, 2011;74:256-61.

[S35] Zhao Y, Chen H, Shang Z, Jiao B, Yuan B, Sun W et al. SD118-xanthocillin X (1), a novel marine agent extracted from *Penicillium commune*, induces autophagy through the inhibition of the MEK/ERK pathway. Mar Drugs, 2012;10:1345-59.

[S36] Wang J, Liu P, Wang Y, Wang H, Li J, Zhuang Y, Zhu W, Antimicrobial Aromatic Polyketides from Gorgonian‐Associated Fungus, *Penicillium commune* 518. Chinese J Chem, 2012;30:1236-42.

[S37] Chen Y, Mao W, Wang J, Zhu W, Zhao C, Li N et al. Preparation and structural elucidation of a glucomannogalactan from marine fungus *Penicillium commune*. Carbohyd Poly, 2013;97:293-9.

[S38] Pivkin MV, Filamentous fungi associated with holothurians from the sea of Japan, off the Primorye coast of Russia. The Biological Bulletin, 2000;198:101-9.

[S39] Wu G, Ma H, Zhu T, Li J, Gu Q, Li D, Penilactones A and B, two novel polyketides from Antarctic deep-sea derived fungus *Penicillium crustosum* PRB-2. Tetrahedron, 2012;68:9745-9.

[S40] Sonjak S, Frisvad JC, Gunde-Cimerman N, Comparison of secondary metabolite production by *Penicillium crustosum* strains, isolated from Arctic and other various ecological niches. FEMS Microbiol Ecol, 2005;53:51-60.

[S41] Bubnova EN, Fungal diversity in bottom sediments of the Kara Sea. Bot Mar, 2010;53:595-600.

[S42] Lu Z, Zhu H, Fu P, Wang Y, Zhang Z, Lin H et al. Cytotoxic polyphenols from the marine-derived fungus *Penicillium expansum*. J Nat Prod, 2010;73:911-4.

[S43] Kerzaon I, Pouchus YF, Monteau F, Le Bizec B, Nourrisson MR, Biard JF, Grovel O, Structural investigation and elucidation of new communesins from a marine‐derived *Penicillium expansum* Link by liquid chromatography/electrospray ionization mass spectrometry. Rapid Commun Mass Sp, 2009;23:3928-38.

[S44] Gonçalves VN, Campos LS, Melo IS, Pellizari VH, Rosa CA, Rosa LH, *Penicillium solitum*: a mesophilic, psychrotolerant fungus present in marine sediments from Antarctica. Polar Biol, 2013;36:1823-31.

[S45] Kambura AK, Mwirichia RK, Kasili RW, Karanja EN, Makonde HM, Boga HI, Diversity of fungi in sediments and water sampled from the hot springs of Lake Magadi and Little Magadi in Kenya. Afr J Microbiol Res, 2016;10;330-8.

[S46] Pang KL, Phylogeny of the marine Sordariomycetes, in Jones EBG and Pang K-L (Eds) Marine fungi and fungal-like organisms. Marine and Freshwater Botany. Walter de Gruyter, Berlin-Boston, 2012, pp 35-47.

[S47] Pang KL, Alias SA, Chiang MW, Vrijmoed LL, Jones EB, *Sedecimiella taiwanensis* gen. et sp. nov., a marine mangrove fungus in the Hypocreales (Hypocreomycetidae, Ascomycota). Bot Mar, 2010;53:493-8.
