# Supplementary material for: The culturable mycobiota of Flabellia petiolata: First survey of marine fungi associated to a Mediterranean green alga
Source: PLoS One. 2017 Apr 20;12(4):e0175941. doi: 10.1371/journal.pone.0175941 (PMC5398637; doi:10.1371/journal.pone.0175941)
Supplement: S2 Table — (DOCX) [file pone.0175941.s005.docx]

**S2 Table. PCR amplification program details.**

| **primers** | | **ACT-512F/ACT-783R** | **Bt2a/Bt2b** | **ITS1/ITS4** | **LR0R/LR7** | **NS/NS4** |
| --- | --- | --- | --- | --- | --- | --- |
| **initial denaturation** | | 95 °C for 10' | 95 °C for 10' | 95 °C for 10' | 95 °C for 10' | 95 °C for 10' |
| **PCR cycle** | **denaturation** | 94 °C for 15'' | 95 °C for 35'' | 95 °C for 1' | 95 °C for 1' | 95 °C for 1' |
|  | **annealing** | 61 °C for 20'' | 58 °C for 20'' | 55 °C for 50'' | 50 °C for 50'' | 50 °C for 50'' |
|  | **elongation** | 72 °C for 40'' | 72 °C for 50'' | 72 °C for 1' | 72 °C for 1.5' | 73 °C for 1.5' |
| **final elongation** | | 72 °C for 10' | 72 °C for 5' | 72 °C for 8' | 72 °C for 10' | 72 °C for 10' |
| **number of cycles** | | 35 | 35 | 35 | 40 | 40 |
| **reference** | | Carbone & Kohn 1999 | Glass & Donaldson 1995 | Schoch et al. 2012 | Lapeyre et al. 1993 | Redou et al. 2016 |
|  |  |  |  | White et al. 1990 |  |  |

PCR primers: ACT-512F/ACT-783R, Bt2a/Bt2b, ITS1/ITS4, LR0R/LR7, NS/NS4, for amplification of actin, β−tubulin, Internal Transcribed Spacer, Large ribosomal SubUnit and Small ribosomal SubUnit sequences, respectively.
